# In vivo PDX CRISPR/Cas9 screens reveal mutual therapeutic targets to overcome heterogeneous acquired chemo-resistance

**DOI:** 10.1038/s41375-022-01726-7

**Published:** 2022-11-04

**Authors:** Anna-Katharina Wirth, Lucas Wange, Sebastian Vosberg, Kai-Oliver Henrich, Christian Rausch, Erbey Özdemir, Christina M. Zeller, Daniel Richter, Tobias Feuchtinger, Markus Kaller, Heiko Hermeking, Philipp A. Greif, Daniela Senft, Vindi Jurinovic, Ehsan Bahrami, Ashok Kumar Jayavelu, Frank Westermann, Matthias Mann, Wolfgang Enard, Tobias Herold, Irmela Jeremias

**Affiliations:** 1 Research Unit Apoptosis in Hematopoietic Stem Cells, Helmholtz Zentrum München, German Research Center for Environmental Health (HMGU), Munich, Germany; 2 Anthropology and Human Genomics, Faculty of Biology, Ludwig Maximilian University (LMU), Martinsried, Germany; 3 Clinical Division of Oncology, Department of Internal Medicine, Medical University of Graz, Graz, Austria; 4 Division of Neuroblastoma Genomics, German Cancer Research Center (DKFZ), Heidelberg, Germany; 5 Hopp Children’s Cancer Center (KiTZ), Heidelberg, Germany; 6 Department of Medicine III, and Laboratory for Leukemia Diagnostics, Department of Medicine III, University Hospital, LMU Munich, Munich, Germany; 7 Department of Pediatrics, Dr. von Hauner Children´s Hospital, University Hospital, LMU Munich, Munich, Germany; 8 Experimental and Molecular Pathology, Institute of Pathology, Ludwig Maximilian University (LMU), Munich, Germany; 9 German Cancer Consortium (DKTK), Partnering Site Munich, Munich, Germany; 10 Department of Proteomics and Signal Transduction, Max Planck Institute of Biochemistry, Martinsried, Germany; 11 Clinical Cooperation Unit Pediatric Leukemia, German Cancer Research Center, Heidelberg, Germany

**Keywords:** Chemotherapy, Cancer therapeutic resistance, Acute lymphocytic leukaemia, Cancer models, Cancer genomics

## Abstract

Resistance towards cancer treatment represents a major clinical obstacle, preventing cure of cancer patients. To gain mechanistic insights, we developed a model for acquired resistance to chemotherapy by treating mice carrying patient derived xenografts (PDX) of acute lymphoblastic leukemia with widely-used cytotoxic drugs for 18 consecutive weeks. In two distinct PDX samples, tumors initially responded to treatment, until stable disease and eventually tumor re-growth evolved under therapy, at highly similar kinetics between replicate mice. Notably, replicate tumors developed different mutations in *TP53* and individual sets of chromosomal alterations, suggesting independent parallel clonal evolution rather than selection, driven by a combination of stochastic and deterministic processes. Transcriptome and proteome showed shared dysregulations between replicate tumors providing putative targets to overcome resistance. In vivo CRISPR/Cas9 dropout screens in PDX revealed broad dependency on *BCL2*, *BRIP1* and *COPS2*. Accordingly, venetoclax re-sensitized derivative tumors towards chemotherapy, despite genomic heterogeneity, demonstrating direct translatability of the approach. Hence, despite the presence of multiple resistance-associated genomic alterations, effective rescue treatment for polychemotherapy-resistant tumors can be identified using functional testing in preclinical models.

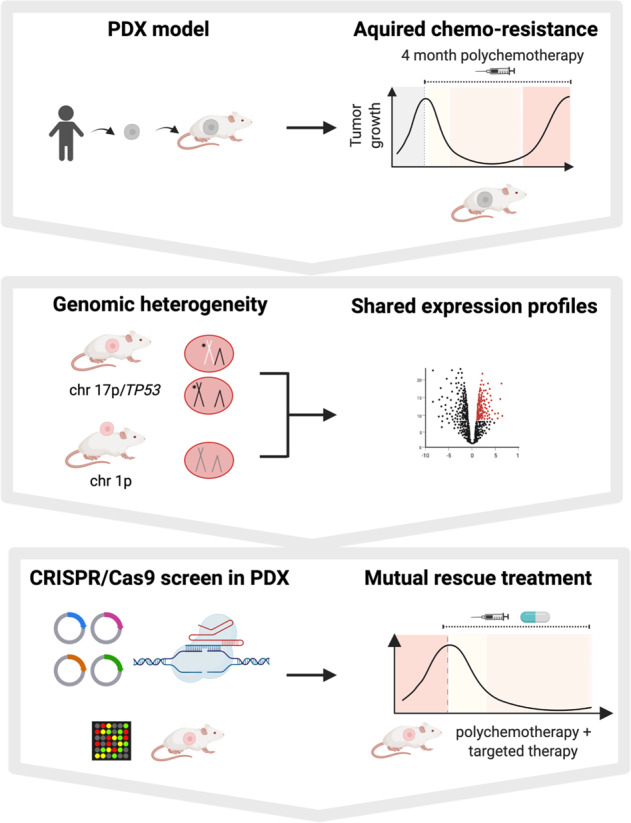

## Introduction

Resistance to anti-cancer therapies represents a highly relevant, unresolved clinical challenge, and continues to be the main cause of cancer-related death [1, 2]. When tumors acquire resistance towards targeted therapies, a single point mutation is frequently found as underlying mechanism [3, 4], while chemotherapy that induces DNA-damage likely acts more diverse. Although conventional chemotherapy has been used for decades, the exact mechanisms underlying acquired resistance remain only partially understood, hindering effective interventions [2, 5].

Large effort has been made to characterize mechanisms responsible for acquired resistance. Sequencing of patients´ tumor cells identified resistance-associated mutations and expression profiles, while single-cell sequencing added tumor heterogeneity and clonal evolution as additional layers of complexity [6–8]. Recent preclinical in vivo studies on targeted therapies detected a sequential pattern of acquired resistance over time, including an intermediate stage of drug tolerance, mimicking the common clinical course in patients [9, 10]. However, similar studies for acquired resistance to polychemotherapy are lacking so far due to technical challenges.

In search for options to investigate and overcome acquired chemo-resistance, we studied patient derived xenograft (PDX) models as they closely mimic the clinical situation and allow integrating phenotypic and in vivo functional levels. Despite their limitations such as clonal skewing, PDX models are considered the most patient-related preclinical model system currently available [11–13], and PDX engraftment times and drug responses were reported to be predictive for patients clinical course [14, 15]. Acute lymphoblastic leukemia (ALL) was used as model disease, where chemotherapy is a crucial treatment component and orthotopic PDX models are amendable to genetic engineering [16]. While PDX models have been used before to study resistance in a number of drugs and tumor entities [9, 10, 17, 18], we advanced the methodology further and added long-term in vivo polychemotherapy, combined with complex genetic engineering. We included widely used alkylating agents, that are often applied over prolonged periods of time in patients, and established a model of acquired resistance resembling the clinical course that was reproducible across different patient samples. Of conceptional and translational importance, our data indicate that prolonged in vivo chemotherapy induces a wide range of different genomic alterations in parallel. In vivo functional genomic screens enabled identification of common vulnerabilities, including *BCL2*, *BRIP1* and *COPS2*, highlighting how rescue treatment can overcome heterogeneous resistance by inhibiting a single target.

## Results

### An in vivo model of acquired resistance in PDX

For a highly patient-related in vivo model, serially transplantable PDX models were used which were derived from pediatric ALL patients (details in Table S1). PDX cells were lentivirally transduced to express recombinant luciferase for highly sensitive and reliable bioluminescence in vivo imaging, to monitor disease progression and treatment effects repetitively in each mouse [16].

We aimed at mimicking a treatment situation in patients, who receive polychemotherapy over prolonged periods of time, which was challenging to establish, as supportive care is unfeasible. The combination of cyclophosphamide (Cyclo) and vincristine (VCR), parts of the polychemotherapy block used to treat lymphoma [19] or adult ALL [20], efficiently reduced tumor load in mice by more than two orders of magnitude and was well tolerated for up to 18 consecutive weeks.

**Fig. 1:**
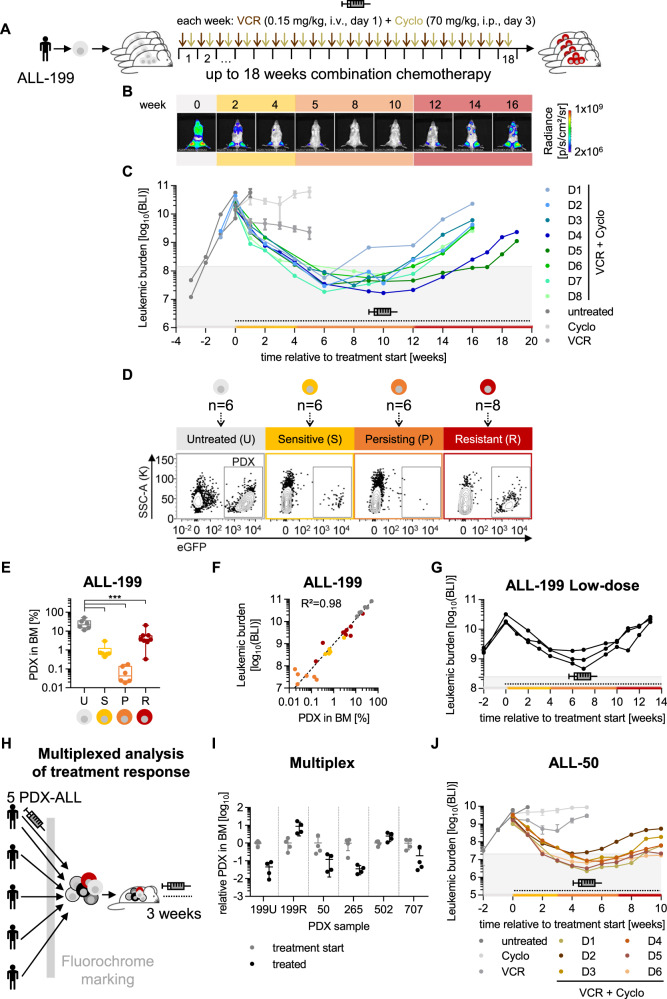
An in vivo model of acquired resistance in PDX ALL. **ALL-199 PDX acquire resistance during long-term treatment in vivo. A** Experimental procedure; PDX ALL-199 cells expressing luciferase were engrafted into 40 mice and in vivo bioluminescence imaging was performed repetitively in each mouse. At high leukemic burden, *n* = 6 mice were sacrificed (time point untreated (U), grey). Remaining mice received weekly injections of either VCR (0.15 mg/kg, i.v.) or Cyclo (70 mg/kg, i.p.) alone (*n* = 3 for Cyclo or VCR, respectively) or in combination (*n* = 28, VCR on day 1 and Cyclo on day 3) for a period of up to 18 weeks. Mice were sacrificed and PDX cells isolated from BM at defined time points: 3 weeks after start of treatment (sensitive (S), yellow, *n* = 6), at minimal residual disease around 10 weeks after start of treatment (persisting (P), orange, *n* = 3 each week, *n* = 6 in total) and at the end of the experiment (resistant (R), red, *n* = 8 in total). Remaining mice (*n* = 8) were sacrificed due to toxicity/illness throughout the experiment. **B** Representative imaging pictures of one mouse (D3) monitored over the course of the experiment. Weeks relative to treatment start are shown. Background colours indicate the disease stage as defined in **A**. **C** Quantification of bioluminescence imaging (BLI) signals. Mice treated with the combination regimen for up to 18 weeks (*n* = 8) were classified as resistant derivatives D1-D8, marked by individual colors. For D1-D8, each dot represents one measurement and each line represents one mouse; for untreated (*n* = 6), Cyclo (*n* = 3) and VCR (*n* = 3) groups mean+/− SD is shown, and individual mice are shown in Fig. S1B. Dashed line and the syringe symbol indicate treatment period; grey area indicates tumor burden below 1% relative to start of treatment. **D** Flow cytometric analysis of PDX cells isolated from BM from one representative mouse at each time point; eGFP marks transgenic ALL-199 cells. **E** Quantification of PDX cells in BM from all mice was measured as in Fig. 1D and is depicted as a boxplot with median, 25th, and 75th percentile, and min/max indicated by whiskers; each dot represents one mouse. ***p < 0.001 by one-way ANOVA followed by Tukey’s multiple comparisons test. **F** Correlation of imaging signals of Figs. 1C and S1B and PDX proportions from Fig. 1E; each dot represents one mouse. Correlation curve and R² were calculated using non-linear regression. **G Low dose treatment of ALL-199**. Experiment was performed as in Fig. 1A except that lower doses of chemotherapy were used (0.1 mg/kg VCR, 50 mg/kg Cyclo). Imaging signals were quantified and are depicted as in Fig. 1C (*n* = 3). **Multiplexed analysis of treatment response of 5 PDX ALL models transplanted into the same mouse**. **H** Experimental procedure. 4 untreated PDX ALL samples (ALL-50, ALL-265, ALL-502, ALL-707) together with untreated ALL-199U and resistant ALL-199R (from Fig. 1D) expressing individual fluorochrome markers were mixed and injected into groups of mice. After 4 weeks of in vivo growth, control mice were sacrificed (treatment start, *n* = 4), and remaining mice received either the combination chemotherapy applied in Fig. 1A–F (treated, *n* = 4, 0.15 mg/kg VCR and 70 mg/kg Cyclo) or solvent (PBS, *n* = 4) for 3 weeks. **I** Fluorochrome expression was analyzed for each mouse by flow cytometry. Individual PDX samples were identified and quantified based on the recombinant molecular markers. Proportion of each PDX sample at the end of treatment was normalized to the mean proportion of the respective sample within the mix at treatment start. Values below 10^0^ upon treatment indicate that the sample responds to treatment; one dot represents the PDX population of one PDX ALL sample within one mouse. **J ALL-50 PDX acquire resistance during long-term treatment in vivo**. Experiment was performed as in Fig. 1A and depicted as in Fig. 1C, except that ALL-50 transgenic for luciferase/mCherry and a genetic barcode were used and mice were treated with adjusted dosing of 0.25 mg/kg VCR and 70 mg/kg Cyclo. Mice were treated with PBS (*n* = 6), VCR (*n* = 3), Cyclo (*n* = 3) or the combination (*n* = 20). Each color marks 1 resistant derivative D1-D6; for D1-D6, each dot represents one measurement and each line represents one mouse; additional data are shown in Fig. S1F–H.

Treatment was initiated at high leukemic burden, shortly before untreated animals would succumb to leukemia (Figs. 1A, B, and S1A). While each drug given alone had minor effects on tumor progression, their combination decreased tumor load by two orders of magnitude within 4 weeks after treatment start, indicating that cells were initially sensitive towards treatment (Figs. 1C and S1B). Thereafter, tumors persisted for several weeks under treatment without growth, a situation resembling the stage of minimal residual disease (MRD) and a drug-tolerant state [16, 21]. Eventually, all tumors resumed growth despite treatment (Fig. 1B, C). The process resembled similar phenotypes described upon targeted therapies [9, 10]. All eight replicate mice showed similar kinetics of acquiring resistance, indicating consistency of the model. As a result, eight resistant ALL-199 derivative PDX models were generated, all originating from the same patient, and referred to as resistant derivatives (ALL-199_D1-D8) from here onwards (Figs. 1C and S1A, B).

For longitudinal sampling, mice were sacrificed at defined time points of untreated (U; at high leukemic burden at the time of treatment start), sensitive (S; 2–3 week after start of treatment), persisting (P; imaging values below 1% relative to treatment start and stable tumor burden) and resistant (R; imaging values increased by at least 10x compared to “P”) tumors (Fig. 1D). The number of re-isolated cells from the murine bone marrow (BM) closely correlated with imaging results (Fig. 1E, F). Development of resistance was reproduced at lower doses where maximum tumor reduction was less pronounced and acquired resistance evolved faster (Fig. 1G).

To test whether the pattern of acquired resistance was conserved across different patient tumors, we searched for a second PDX ALL model with high drug sensitivity. We established a multiplex in vivo approach to estimate therapy response of several PDX models in the same animals and to take advantage of identical treatment conditions and limited resources (Fig. 1H). 6 distinct PDX models obtained from 5 different patients were molecularly labelled, mixed, and mutually injected into the same group of mice (Fig. 1H, Table S1). From post mortem flow cytometry data, growth kinetics and treatment response of each PDX model was calculated (Figs. 1I and  S1C–G). While ALL-502 showed intrinsic treatment resistance, ALL-50 and ALL-265 displayed major treatment response in this multiplex setting and were selected for further studies (Fig. 1I). To achieve tumor reduction by two orders of magnitude, drug doses had to be increased for both PDX models, while drug schedule and route of administration remained unchanged. Upon adjusted dosing, the sequential development of resistance was recapitulated, and 6 resistant derivatives of ALL-50, ALL-50_D1-D6 were established (Figs. 1J and S1H–J). However, experiments had to be terminated early due to toxicity, and in case of ALL-265 even before resistant derivatives could be established (Fig. S1K).

Taken together, long-term polychemotherapy with Cyclo and VCR in vivo led to reliable development of acquired therapy resistance in three phases, across PDX models from different patients, replicate mice, and different doses of polychemotherapy.

### Acquired resistance is leukemia cell-intrinsic and stable

**Fig. 2:**
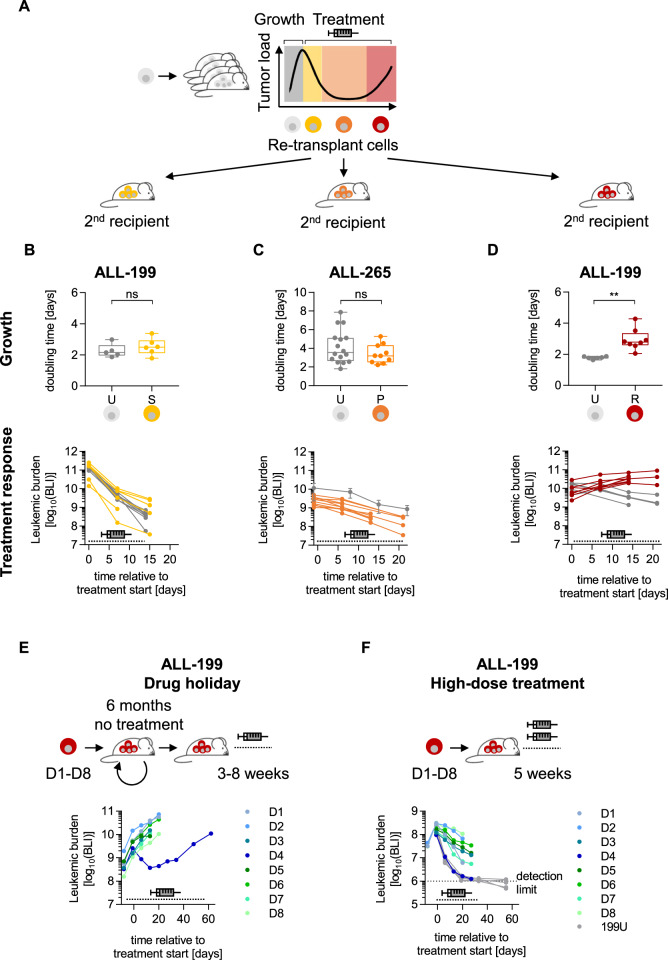
Acquired resistance is leukemia cell-intrinsic and stable. **Resistance phenotype is preserved upon re-transplantation A** Cells isolated at disease stages untreated (grey), sensitive (yellow), persisting (orange), and resistant (red) were re-transplanted into secondary recipient mice. Tumors were allowed to grow for 30–40 days in vivo before treatment was initiated at the same dose, route, and schedule as in the previous passage for 2–3 weeks. Leukemic growth and treatment response was monitored by repetitive imaging. **B** ALL-199S (*n* = 6) from Fig. S2B that were previously treated with combination chemotherapy (0.5 mg/kg VCR; 100 mg/kg Cyclo) for 2 weeks and untreated ALL-199 (*n* = 5) were used. Data is also shown in the middle panel of S2B. **C** ALL-265P (*n* = 10) from Fig. S1I that were previously treated with combination chemotherapy (0.3 mg/kg VCR; 70 mg/kg Cyclo) for at least 7 weeks were used. Untreated ALL-265U (*n* = 16) shown in Fig. S1I were used as control (depicted in grey). **D** ALL-199R (*n* = 8) from Fig. 1A–F previously treated with combination chemotherapy for up to 18 weeks (0.15 mg/kg VCR, 70 mg/kg Cyclo) and ALL-199U untreated controls (*n* = 6) were used. **B-D:** *Upper panels:* doubling time was calculated based on imaging values. Box indicates median, 25th, and 75th percentile; whiskers indicate min/max; each dot represents one mouse. **p < 0.01 by unpaired t-test. ns: not significant. *Lower panels:* treatment response was monitored by repetitive imaging; each dot represents one measurement and each line represents one mouse except for ALL-265U in C, where mean+/− SD is shown. **E Resistance remains stable after drug holiday**. Resistant derivatives D1-D8 were each transplanted into one mouse, grown to high tumor burden, and each re-transplanted into one next recipient mouse for 3 passages, resulting in a total of 6 months in the absence of treatment. At 4th passage and upon advanced tumor load, mice were treated with combination chemotherapy (0.15 mg/kg VCR, 70 mg/kg Cyclo) used in Fig. 1A–F for 3–8 weeks; data are depicted as in Fig. 1C; each dot represents one measurement and each line represents one mouse. **F Partial resistance upon high-dose treatment**. Resistant ALL-199 derivatives D1-D8 (*n* = 1 each) and untreated control cells (ALL-199U; *n* = 5) were each transplanted into mice; upon advanced tumor load, mice were treated with high-dose combination chemotherapy (0.6 mg/kg VCR, 100 mg/kg Cyclo) for 5 weeks and treatment response was monitored by repetitive imaging; mice engrafted with previously untreated cells were monitored for 5 more weeks to assess putative leukemia re-growth; data are depicted as in Fig. 1C, each dot represents one measurement and each line represents one mouse.

To characterize the resistance phenotype further, we re-challenged PDX cells from the distinct stages with polychemotherapy (Fig. 2A). When PDX ALL-199 and ALL-265 cells were re-transplanted into secondary recipient mice, growth rates of sensitive (Figs. 2B and S2A–C) and persistent (Figs. 2C and S2D) PDX ALL cells were not altered compared to control cells, i.e., untreated cells from the respective PDX sample, in line with our previous studies [16].

When treatment response of re-transplanted cells was compared, sensitive (Figs. 2B, S2A–C) and persisting cells (Figs. 2C, S2D) responded like the respective untreated control cells in both PDX models. Both ALL-199S and ALL-265S remained sensitive even when cells were exposed to short term treatment over 2-3 weeks in 3 consecutive passages (Figs. S2A–C).

In contrast, resistant ALL-199R cells expanded significantly slower in secondary mice (Figs. 2D and S2D), indicating that emergence of resistance was accompanied by decreased proliferative fitness, as observed in models that develop resistance to nucleotide analogues [22]. Additionally, response to polychemotherapy was strongly diminished in resistant ALL-199R and resistant ALL-50R derivatives compared to respective control PDX cells, that received treatment for the first time (Figs. 2D, S2E). Both PDX models expanded in mice despite continuous therapeutic pressure, indicating that resistance was stably established in these derivatives. In contrast, pre-treatment of mice to condition the bone marrow niche did not alter engraftment, growth, or treatment response of ALL-199 PDX cells (Figs. S2F–H). The data indicate that cells acquired a leukemia cell-intrinsic, stable resistance, which most likely developed late during the persisting stage.

To test whether resistance might persist over prolonged periods of time in the absence of selection pressure, eight resistant ALL-199R derivatives D1-D8 were grown in mice in the absence of therapy for 4 passages or 6 months of “drug holiday” until treatment was resumed (Figs. 2E, S2I). 7 of 8 derivatives maintained resistant against polychemotherapy, indicating that irreversible resistance had developed. As exception, D4 showed a partial and less stable phenotype (Figs. 2E, S2J).

To understand the strength of the resistant phenotype, mice were challenged with higher doses of both drugs, tolerable for short periods of time. Mice bearing ALL-199U cells were cured by this chemotherapy regimen, while response to treatment was clearly decreased in the majority of resistant derivatives, although at varying extents with derivative D4 displaying a partial and dose-dependent resistance (Fig. 2F).

Overall, we have established an in vivo model where different derivatives of the same PDX sample adopted a heterogeneous pattern of acquired chemo-resistance.

### Heterogeneous genomic alterations emerge in PDX with acquired resistance

**Fig. 3:**
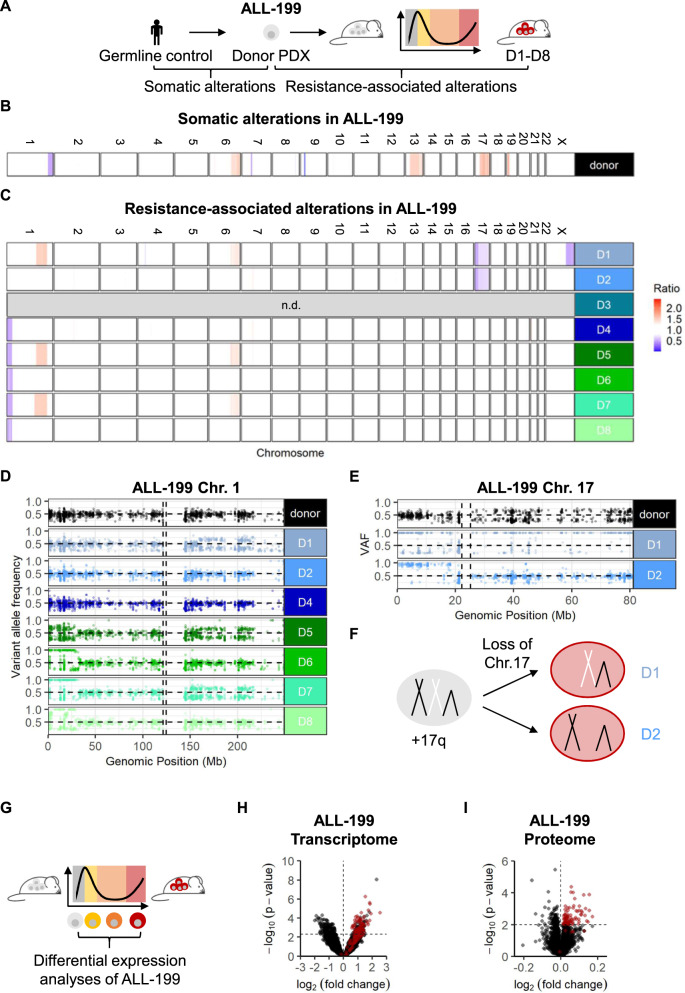
Heterogeneous genomic alterations, but shared expression features upon acquired resistance in ALL-199. **Genomic characterization of resistant ALL-199 derivatives A** Whole exome sequencing was performed on ALL-199 germ line control obtained from healthy BM cells of the patient, donor PDX model, untreated cells and the eight resistant derivatives (D1-D8). Comparison between donor ALL-199 PDX and germline control to identify somatic alterations of the PDX leukemia (**B**); comparison between resistant D1-D8 and donor PDX to identify resistance-associated alterations (**C**); losses are depicted in blue and gains in red. Each row depicts one chromosome and each line depicts one sample. D3 was excluded due to poor sequencing quality. Magnification into chr. 1 (**D**) and chr. 17 (**E**); each dot represents a SNV as determined using hg19 as reference; homozygous SNV (VAF > 0.8) present in the donor sample and SNV with VAF < 0.2 were excluded. Horizontal dashed line represents VAF = 0.5, vertical dashed lines represent centromere positions. **F** Schematic representation of chr. 17 in donor PDX ALL-199 (light grey), where one allele of chr. 17q is duplicated, and in resistant derivatives D1 and D2 (red), where one of two whole chr. 17 alleles were lost. **G-I Transcriptomic and proteomic profiling G** Experimental layout: Transcriptomes were determined from ALL-199U (*n* = 5), ALL-199S (*n* = 6), ALL-199P (*n* = 5) and ALL-199R (*n* = 24, combined samples shown in Figs. 1C, 2D, S2I). Proteomes of ALL-199U (*n* = 4) and ALL-199R (*n* = 8) were analyzed. **H** Volcano plot representing genes differentially expressed between ALL-199U and ALL-199R; each dot represents one transcript; dashed lines indicate cut-offs for significant up- or downregulation (*p* < 0.005 and log_2_ fold-change >0 or <0, respectively). Red dots indicate candidates, which were chosen for further analysis. **I** Volcano plot of all differentially expressed proteins between ALL-199U and ALL-199R is shown and depicted as in Fig. 3H; dashed lines indicate cut-offs for significant up- or downregulation (*p* < 0.01 and log_2_ fold-change >0 or <0, respectively).

As resistance was found to be a stable trait, we hypothesized that genomic alterations in leukemia cells might underlie the resistant phenotype and performed whole exome sequencing. For ALL-199, untreated ALL-199U, resistant derivatives ALL-199_D1-D8 and a germline control were analyzed (Fig. 3A). Data for ALL-199_D3 had to be excluded due to insufficient sequencing quality.

Analysis of ALL-199 revealed that in addition to the known germline trisomy 21 and the somatic P2RY8-CRLF2 fusion and a loss of CDKN2A (Table S1), somatic copy number alterations (CNA) on chromosomes (chr.) 1, 6, 7, 9, 13, 14, 17 and 19 were detected in the untreated PDX model (Figs. 3B, S3, Table S2). When resistant derivatives ALL-199_D1-D8 were compared to untreated PDX to determine resistance-associated alterations, four additional large CNA became apparent on chr. 1, 6, 17, and X, which were mostly recurrent, but present in individual combinations in each derivative (Figs. 3C and S3). Chromosomal alterations including a partial loss of the short arm of chr. 1 and the loss of chr. 17 were mutually exclusive in ALL-199_D4-D8 and ALL-199_D1-D2, respectively. Of note, chr. 17q was amplified in the donor sample, and genes located on 17p had two copies, while genes on 17q had three copies in the untreated sample. After the loss of one whole chromosome 17, only one copy of 17p and two copies of 17q are left in ALL-199_D1 and D2.

A similar analysis was performed in ALL-50, where barcoded ALL-50 donor, untreated ALL-50U, and 6 resistant derivatives, ALL-50_D1-D6 were analyzed (Fig. S4A). WES data for ALL-50_D6 had to be excluded due to insufficient sequencing quality. Barcode analysis revealed that in ALL-50, one single clone clearly dominated in 5 of 6 resistant ALL50 derivatives after long-term polychemotherapy (Fig. S4B); one resistant sample, ALL-50_D3 displayed more heterogeneous selection with 4 distinct predominant barcodes (Fig. S4B). Analysis of CNA in ALL-50 revealed multiple small CNAs in ALL-50_D1-D5 compared to the donor sample, e.g., on chr. 2, 16, 21 (Figs. S4C, D and Table S3). Similar to our observation in ALL-199, CNA acquired during development of resistance were heterogeneous across ALL-50 resistant derivatives, despite that the majority of samples originated from the same subclone (Figs. S4B–D, Table S3).

In order to describe the presence of subclonal CNA in ALL-199, variant allele frequencies (VAFs) of common single nucleotide polymorphisms (SNP) located on chromosomes 1 and 17 were analyzed in detail (Fig. 3D, E, and S5A). While a clonal loss was detected in ALL-199_D6-D8, subclonal losses were observed in ALL-199_D4 and D5 (Fig. 3D), further reflecting the heterogeneity in clonal composition of individual derivatives. Analysis of previously trisomic chr. 17 indicated that different alleles were lost in ALL-199_D1 and D2 (Fig. 3E, F, and S5A). Interestingly, the tumor suppressor *TP53* is located on the short arm of chromosome 17 and both derivatives, ALL-199_D1 and D2, carried distinct mutations on the single remaining allele of *TP53* at VAFs of 95% and 82%, respectively, indicating hemizygous mutations. In ALL-199_D1, a frame-shift mutation was detected in the frequently mutated DNA binding domain [23], in ALL-199_D2, a splice site mutation was detected in exon 3 (Fig. 3E,  S5B and Table S4). As both *TP53* mutations were not detectable in the donor sample at a coverage of 3,204 or 8,233 reads, respectively (Table S4), these mutations were infrequent before treatment. Moreover, *TP53* point mutations were also found in ALL-50_D2 and D5 (Fig. S6, Table S5). Given the long-known link between p53 inactivation and chemo-resistance [23–26], it is likely that p53 loss of function contributes to resistance in ALL-199_D1 and D2 and in subclones of ALL_50_D2 and D5.

In ALL-199_D4-D8, recurrent deletions of 1p were observed, which are frequently seen in diverse cancers [27], and have been associated with poor prognosis in neuroblastoma [28], breast cancer [29], colon cancer [30], and myeloma [31]. To analyze whether 1p deletions may be involved in resistance to chemotherapy in our model, we determined the smallest region of overlapping deletions (SRO) for ALL-199_D4-D8, which mapped to 1p36, a region that contains dosage-dependent tumor suppressor genes [32], whose downregulation might contribute to resistance. To analyze differences in gene expression of 1p-deleted vs. 1p non-deleted resistant derivatives, transcriptome analysis of untreated, sensitive, persisting and resistant cells was performed (Fig. 3G), and resistant samples ALL-199_D1-D2 were compared to 1p-deleted ALL-199_D4-D8 (Fig. S5C, D). Of the top 22 differentially expressed genes mapping to the 1p36 SRO, 21 were downregulated in resistant derivatives affected by the deletion (Fig. S5C, D Tables S6, S7), including the dosage-dependent tumor suppressor candidate *KIF1B* [32] and *ENO1*, an essential gene whose downregulation generates a therapeutic vulnerability in 1p36 deleted cancers [33, 34]. This indicates that downregulation of 1p36 genes in derivatives ALL-199_D4-D8 may contribute to resistance development. Interestingly, *RPL22*, which was found recurrently mutated in all 5 ALL-50 resistant derivatives analyzed is located on 1p36 (Fig. S6 and Table S5), suggesting an important role of 1p36 genes in resistance development in both PDX samples.

In sum and reminiscent of similar observations described in primary clinical samples [26, 35], despite the common phenotype of chemo-resistance, the different derivatives of both ALL PDX samples harbor distinct but recurrent chromosomal and genomic alterations.

### Shared altered expression features upon acquired resistance

To decipher whether common characteristic of resistance might exist despite different genomic alterations, we further analyzed ALL-199. The transcriptome analysis of untreated, sensitive, persisting and resistant cells (Fig. 3G) revealed a total of 525 genes as significantly deregulated between resistant and untreated ALL-199U cells, with 273 genes significantly upregulated and 252 significantly downregulated (Figs. 3G, H, S7A and Table S7). In addition, proteome was analyzed from resistant derivatives and ALL-199U; Among 171 proteins significantly deregulated, 85 proteins were significantly upregulated in resistant samples, most of them consistently across all derivatives (Figs. 3I, S7B, C and Table S8); due to the low cell numbers retrieved from animals at the persisting stage, proteome analysis was not feasible in persisting ALL-199P. Like transcriptome, proteome clearly distinguished untreated from resistant samples (Figs. S7B, C). Gene set enrichment analysis of transcriptome (Table S9) or proteome (Table S10) indicated processes like cell adhesion, epithelial-mesenchymal transition, p53 pathway, or hypoxia enriched in ALL-199R compared to ALL-199U, albeit not to a statistically significant level. In summary, expression profiling indicated common deregulated genes and proteins in the 8 resistant ALL-199 derivatives, reflecting the mutual resistant phenotype, in the context of heterogeneous genomic changes.

### A CRISPR/Cas9 dropout screen in PDX ALL models in vivo

Common gene expression changes across genomically heterogeneous derivatives might represent a therapeutic opportunity to overcome acquired resistance to polychemotherapy. To test whether individual deregulated genes in ALL-199 can re-sensitize resistant derivatives to therapy, we applied CRISPR/Cas9 dropout screens, using a protocol that we had recently optimized to be performed in vivo in PDX models.

Within the candidates upregulated in transcriptome and/or proteome of ALL-199 (Fig. 3G, H), 223 protein-coding genes were examined in the screen (Table S11). Five sgRNAs were selected per target gene and complemented with positive and negative controls, resulting in a library size of 1196 sgRNAs. The library was cloned into a lentiviral vector that co-expressed selection markers for magnetic and flow cytometric enrichment (Fig. S8A).

**Fig. 4:**
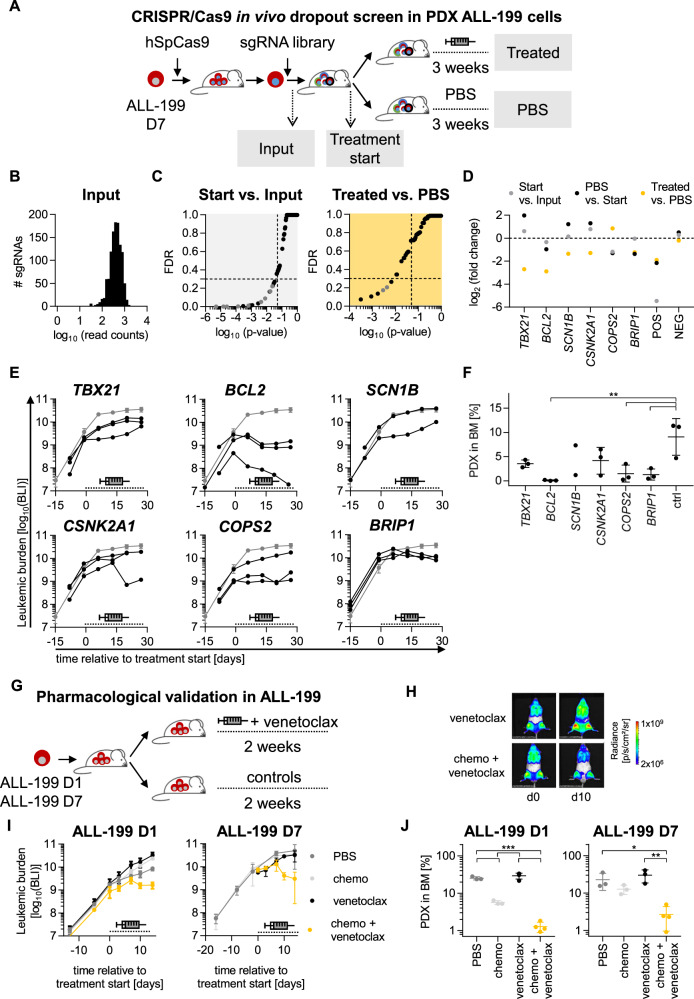
CRISPR/Cas9 induced knockout of *BCL2* or treatment with venetoclax re-sensitize resistant PDX models to treatment. **CRISPR/Cas9** ***in vivo*** **dropout screen in ALL-199 PDX cells reveals candidates relevant for acquired resistance A** Resistant D7 cells were lentivirally transduced to express hSpCas9 followed by transduction with the sgRNA library (Table S11); after 3 days of in vitro cultivation and magnetic cell enrichment, the input sample was collected, and remaining cells transplanted into mice (*n* = 9). After 4 weeks of in vivo growth, control mice were sacrificed (treatment start, *n* = 2) and remaining mice were treated for 3 weeks with the combination chemotherapy (treated, *n* = 4, VCR 0.15 mg/kg, Cyclo 70 mg/kg) as in Fig. 1A–F or PBS (*n* = 3). PDX cells were re-isolated from murine BM of all mice, genomic DNA isolated, sgRNA library amplified, and sequenced by NGS, followed by data analysis using the MAGeCK algorithm. **B** sgRNA distribution of the input sample. **C** MAGeCK results comparing start vs. input (left panel) or treated vs. PBS (right panel); one dot represents one gene (pooled analysis of 5 sgRNAs per gene); grey dots indicate positive controls with *p* < 0.05; dashed lines indicate cut-off of *p* = 0.05 and false discovery rate (FDR) = 0.3. **D** Top dropouts from the screen plus controls as defined by either *p* < 0.05 and FDR < 0.3 in screen on D7 (*TBX21*, *BCL2*, *SCN1B*, *CSNK2A1, and BRIP1*) or by consistent depletion in the two screens on D7 and D5 (*COPS2*). **Single validation of targets identified by CRISPR/Cas9 screen. E** Experiments were performed identically as described for Fig. 4A, except that a single sgRNA was transduced per sample and mouse, targeting one of the six candidates depicted in Fig. 4D or non-targeting controls (19 different sgRNAs in total, i.e., 3 sgRNAs per target gene and 1 non-targeting control sgRNA). Cells were transplanted into 21 mice (*n* = 1 per target sgRNA, *n* = 3 per target gene or control). At high leukemic burden, all mice were treated with combination therapy used in Fig. 1A–F (0.15 mg/kg VCR, 70 mg/kg Cyclo) for 3 weeks and treatment response was monitored by repetitive imaging; each black line represents a single mouse, the grey line shows mean+/− SD of the 3 mice harboring non-targeting control sgRNA; the dashed line indicates the treatment period. **F** At the end of the experiment, PDX ALL-199 cells were isolated from the murine BM and leukemic burden quantified using flow cytometry and proportion of PDX was calculated. Mean+/− SD for each group is shown. One dot represents one mouse. **p < 0.01 by one-way ANOVA followed by Tukey’s multiple comparisons test. **G-J Venetoclax re-sensitized resistant ALL-199 PDX derivatives to treatment. G** Experimental design: ALL-199 D1 or D7 cells were engrafted into groups of NSG mice. At high leukemic burden, mice were treated for 2 weeks with PBS alone (*n* = 3) or venetoclax alone (*n* = 4, 100 mg/kg p.o. days 1–5) or the combination chemotherapy plus PBS (*n* = 3) or the combination chemotherapy plus venetoclax (*n* = 4, venetoclax 100 mg/kg p.o. days 1–5, 0.15 mg/kg VCR i.v. day 3 and 70 mg/kg Cyclo i.p. day 5). **H** Treatment response was monitored by imaging; One representative mouse per group is shown at d0 and d10 of treatment, respectively. **I** Quantification of imaging signals from all mice analyzed; mean+/− SD per group is shown; dashed line indicates treatment period; for D7, data of PBS and treated group was derived from previous experiment for comparison (Fig. S8E). **J** At the end of the experiment, PDX ALL-199 cells were isolated from the murine BM and quantified using flow cytometry and proportion of PDX was calculated. Mean+/− SD for each group is shown. One dot represents one mouse (*n* = 3 for PBS and treated, *n* = 4 for venetoclax and treated + venetoclax). *p < 0.05, **p < 0.01, ***p < 0.001 by one-way ANOVA followed by Tukey´s multiple comparisons test.

Two derivatives, ALL-199_D7 and ALL-199_D5, were selected and lentivirally transduced to express recombinant Cas9 and the sgRNA library (Figs. 4A and S8A, B). The screen was performed under strict quality controls (Figs. 4B and S8C–E). Mice were treated with chemotherapy or control and library composition was compared between both treatment arms, using data from ´input´ before cell transplantation and ´treatment start´ before onset treatment as additional controls.

The sgRNAs that dropped out in the ´input´, ´treatment start´ or ´untreated´ samples were categorized as essential genes required for leukemic engraftment, growth, and survival; these sgRNAs dropped out along with the essential control genes (Fig. 4C left, S8F and Table S12). Only dropouts found exclusively in treated, but not in untreated tumors were categorized as genes sensitizing for treatment (Fig. 4C right). From the strongly depleted genes in D7, *TBX21*, *BCL2*, *SCN1B CSNK2A1*, and *BRIP1* dropped out exclusively under treatment pressure. *COPS2* dropped out in both screens performed in D7 and D5 (Fig. 4D and S8G–I). Neither of the dropout genes were found to bear a mutation affecting protein function, but all were significantly upregulated either on RNA (TBX21, SCN1B, CSNK2A1, BRIP1) or protein level (BCL2) or both (COPS2).

Overall, the CRISPR/Cas9 PDX in vivo screen detected multiple dropout candidates with putative essential and/or treatment related function.

### Knockout of *BCL2* sensitizes resistant PDX cells towards chemotherapy

All 6 candidate hits from the dropout screens in D5 and D7 were validated by testing one single sgRNA per animal and 3 sgRNAs per gene in vivo in D7. Cells lentivirally transduced with Cas9 and individual sgRNAs were injected into mice and treated with the polychemotherapy regimen at high leukemic burden for 3 weeks. Tumor load was monitored by imaging and percentage of PDX cells in BM determined at the end of the experiment (Fig. 4E, F). In these in vivo validation experiments and before onset of treatment, leukemic growth was comparable across cells transduced with the individual sgRNAs, confirming the non-essential role of all genes (Fig. 4E). As most obvious hit, treatment strongly reduced tumor load in all 3 samples bearing *BCL2*-sgRNAs, indicating that knockout of *BCL2* restored sensitivity of resistant PDX ALL-199 cells towards treatment in vivo. Similarly, knockout of *COPS2* or *BRIP1* achieved significant reduction of tumor burden. sgRNAs against all other candidates showed a similar trend, in general confirming the results of the CRISPR/Cas9 in vivo screen, but with less consistent and/or less pronounced phenotypes that did not reach significance. Taken together, knockout of *BCL2*, *COPS2* or *BRIP1* showed the strongest phenotype and re-sensitized resistant ALL-199R cells towards in vivo treatment.

### Venetoclax sensitizes resistant derivatives with different genomic alterations to chemotherapy

To translate the molecular insights into a clinic-related setting, the pharmacological BCL2 antagonist venetoclax was studied as proof-of-concept, as it is (i) increasingly applied in patients, especially in situations of resistance against conventional chemotherapy [36, 37] and (ii) high BCL2 expression correlated to inferior treatment response in both B-ALL (*p* < 0.01) and AML (Fig. S9A, B) [38–40]. Mice-bearing resistant D7 derivatives were treated with the polychemotherapy regimen in combination with venetoclax (Fig. 4G). In analogy to *BCL2* knockout, venetoclax alone did not alter tumor growth, whereas the combination of venetoclax plus chemotherapy clearly decreased tumor load, indicating the potential of venetoclax to overcome treatment resistance to polychemotherapy (Fig. 4H, I and S9C, D).

To evaluate whether targeting BCL2 can overcome resistance independent from the genomic alterations that have been acquired under treatment (Fig. 3), we repeated the experiment with derivative D1. Resistance to chemotherapy in D1 is likely driven by the loss of functional p53. In agreement with a reported p53-independent mechanism of action of venetoclax [41] and similar to D7, venetoclax re-sensitized D1 towards polychemotherapy (Fig. 4J). Thus, co-treatment of polychemotherapy plus venetoclax re-sensitized genomically diverse derivatives to chemotherapy, demonstrating translatability of the data obtained in PDX.

Taken together, long-term treatment of mice with a combination therapy of cyclophosphamide and vincristine-induced acquired resistance accompanied by individual mutations and CNAs between replicate tumors. Similar changes in transcriptome and proteome enabled identifying BCL2, COPS2, and BRIP1 as common targets to re-sensitize different derivatives towards chemotherapy, despite underlying genomic heterogeneity.

## Discussion

We established a preclinical in vivo model of acquired resistance using long-term treatment of mice with a combination therapy of cyclophosphamide and vincristine. Resistance evolved with a surprisingly similar kinetic across replicate mice in the PDX models tested and represented a stable feature in cells that grew under treatment, but not in persisting precursors. Acquired chemo-resistance was associated with genomic heterogeneity, with individual mutations and CNAs in common chromosomal regions between replicate tumors that evolved in parallel and independently. Our model might mimic mechanisms evolving during the clinical course of patients acquiring chemo-resistance [8]. The complexity identified requires an effective rescue therapy capable of combating a wide range of resistance-associated changes. We show here that targeting BCL2, COPS2 or BRIP1 might carry this capability.

Eight derivatives of ALL-199 and six derivatives of ALL-50 evolved in the respective PDX model of single patient´s tumors in vivo. All derivatives acquired distinct individual genomic alterations during treatment, indicating independent evolutionary processes, which converged in their resistance-inducing effect, reflecting intra-tumor heterogeneity. Of note, identical alterations were described to be associated with reduced treatment response and inferior prognosis in clinical studies [23, 24, 31, 32].

Because numerous genomic alterations were non-detectable in untreated cells, they have likely developed de novo during chemotherapy in ALL-199. At least two independent genomic mechanisms evolved to induce chemo-resistance in ALL-199, located either on chr. 1 or 17, while remaining CNAs might mark different cells of origin. As underlying concept, deterministic processes might have guided alterations towards chr. 1 and 17, while stochastic processes might have occurred at these sites, leading to individual genetic alterations in each derivative. While alterations in both chr. 1 and 17 have been described before as associated with acquired chemo-resistance [23, 25, 26, 32], we show here that a single tumor developed both of them in parallel, as combination of chromosomal alterations and mutations. In line, resistant ALL-50 derivatives also showed heterogeneity in the clonal composition, albeit barcode analysis indicated an identical clone of origin. In addition, acquired heterogeneous CNA and mutations suggest intra-tumor heterogeneity during development of resistance. It will be interesting to use longitudinal sampling and single-cell sequencing of primary tumor cells to determine whether parallel evolution of different CNAs also occurs in patients under chemotherapy.

Resistance-associated genomic heterogeneity limits the options for second-line treatment, as addressing single alterations in a mutation-targeting approach likely fails to eradicate the entire tumor. As alternative, treatment might be selected based on expression profiles to combat heterogeneous resistance-associated alterations. Our model is of major importance to capture and address the impact imposed by intra-tumor heterogeneity on acquired resistance and rescue treatment, putatively important across numerous tumor entities [42, 43].

Using in vivo CRISPR/Cas9 knockout screening, several putative targets were identified which might be capable to re-sensitize resistant derivatives of ALL-199 towards chemotherapy, including *BCL2, COPS2* and *BRIP1*. Translational relevance of our results is supported by clinical data, where venetoclax and other BH3 mimetics are increasingly used in combination treatment in a wide range of different tumors [36, 37], including resistant ALL (NCT03808610, NCT03319901, NCT03504644). The cancer susceptibility gene *BRIP1*, as well as COPS2, part of the COP9 signalosome, have both been implicated in DNA damage repair [44, 45], and were associated with treatment failure and dismal prognosis [46–48]. As *BRIP1* is a known regulator of response to cytotoxic agents [49], and *COPS2* dropped out mutually in both our CRISPR/Cas9 screens, analyzing their role in treatment resistance warrants further evaluation.

In summary, we demonstrate that acquired resistance against chemotherapy is accompanied by multiple different genetic alterations occurring independently and in parallel, and identify novel candidate genes capable to overcome heterogeneous mechanisms of resistance.

## Materials and methods

### Methods described in the supplement

Details are provided in the supplement regarding establishment and serial transplantation of transgenic PDX models, monitoring of tumor burden during in vivo growth, in vivo drug treatment regimens, sequencing of exome and transcriptome, proteome analysis, execution of the CRISPR/Cas9-dropout screen and statistical analysis.

## Supplementary information

Supplemental Information

Supplemental Tables

## Data Availability

Transcriptome data generated in this study are publicly available in Gene Expression Omnibus (GEO) at GSE214447. Proteome data generated in this study are publicly available in Proteomics Identification Database (PRIDE) at PXD036517. Whole Exome Sequencing raw data generated in this study are not publicly available due to information that could compromise patient privacy or consent but are available upon reasonable request from the corresponding author.
