# Supplementary material for: In vivo PDX CRISPR/Cas9 screens reveal mutual therapeutic targets to overcome heterogeneous acquired chemo-resistance
Source: Leukemia. 2022 Nov 4;36(12):2863–74. doi: 10.1038/s41375-022-01726-7 (PMC9712105; doi:10.1038/s41375-022-01726-7)
Supplement: Supplementary file 1 — Supplemental Information [file 41375_2022_1726_MOESM1_ESM.pdf]

Supplemental Information

for

In vivo PDX CRISPR/Cas9 screens reveal mutual therapeutic targets to overcome heterogeneous acquired chemo-resistance

Anna-Katharina Wirth[1], Lucas Wange[2], Sebastian Vosberg[3], Kai-Oliver Henrich[4,5], Christian Rausch[6], Erbey Özdemir[1], Christina M. Zeller[1], Daniel Richter[2], Tobias Feuchtinger[7], Markus Kaller[8], Heiko Hermeking[8], Phillip A Greif[6,9], Daniela Senft[1], Vindi Jurinovic[1,6], Ehsan Bahrami[1], Ashok Kumar Jayavelu[10,11], Frank Westermann[4,5], Matthias Mann[10], Wolfgang Enard[2], Tobias Herold[1,6], Irmela Jeremias[1,7,9,☆]

This pdf contains

Supplemental Methods and References

Supplemental Tables

Supplemental Figures and Legends

Supplemental Methods

Establishment and serial transplantation of transgenic patient derived xenograft (PDX) models

Eight to 16 weeks old male and female NOD.Cg-*Prkdc*^{scid} *Il2rg*^{tm1Wjl}/SzJ (NSG) mice (The Jackson Laboratory, Bar Harbour, ME, USA) were kept in individually ventilated cages (IVCs). The animal rooms were fully air-conditioned with a temperature of 20-24°C and 45-65% humidity according to Annex A of the European Convention 2007/526 EC. The maximum stocking density of the cages corresponds to Annex III of the 2010/63 EU. The cages were constantly filled with structural enrichment and the animals had unlimited access to food and water. The animals were inspected daily and scored weekly. Signs of monitoring were a body condition score (BCS), posture and movement, behavior, condition of care, eyes and breathing. Humane endpoints were a BCS of <1, marked lameness or inability to move. Animals with apathetic behavior and no reaction to external stimuli, as well as swollen eyes or swollen peritoneal area and gasping also lead to a termination. In therapy trials, loss of body weight beyond 15% for more than two days or beyond 20% relative to day of treatment start resulted in termination. Establishing serially transplantable ALL PDX models in NSG mice from patient material, re-isolating PDX cells from murine spleen and bone marrow (BM), PDX cell culture, lentiviral transduction, enrichment of transgenic cells and *in vivo* bioluminescence imaging (BLI) were performed as described previously (1-5). In brief, up to 1 x 10^7 fresh or thawed transgenic PDX ALL cells were injected into the tail vein of mice. To monitor engraftment, tumor growth and treatment response, tumor burden of mice was regularly assessed by IVIS Lumina II (Caliper) with Living Image version 4.4 software (PerkinElmer) starting at day 14 after transplantation and additionally, 50µl peripheral blood (PB) was repetitively collected from the tail vein starting at day 28 after transplantation. PB was analyzed by flow cytometry after staining for murine CD45 and human CD38 (2). At advanced leukemic disease, at defined time-points or at signs of clinical disease (rough fur, hunchback, reduced motility, paralysis) mice were sacrificed by exposure to CO_2 or cervical dislocation and ALL PDX cells were re-isolated from murine BM and/or spleen for further analyses.

In vivo treatments

Mice were treated systemically with vincristine (VCR) i.v. once per week and/or with cyclophosphamide (Cyclo) i.p. once per week and/or with venetoclax (ABT-199, SelleckChem) 100 mg/kg p.o. five times per week. In all combination treatment experiments, VCR was applied two days prior to Cyclo; In combination treatment experiments of all three drugs, venetoclax was applied on days 1-5, VCR on day 3 and Cyclo on day 5 of each treatment week. VCR and Cyclo were diluted in sterile PBS for efficient application, ABT-199 was dissolved in 1% Carboxymethyl cellulose and 5% DMSO. Animals of the control groups were treated with solvent (PBS) by the same route of administration. Drug concentrations were calculated from clinically relevant concentrations converting the human doses to mouse equivalent doses based on body surface area and differences in metabolism as described (2, 6, 7).

Drug dosages of VCR and Cyclo were further optimized for each PDX sample individually to ensure tumor reduction by at least 2 orders of magnitude: parental ALL-199 and mice in the multiplex or pretreatment experiment received 0.15 mg/kg VCR and 70 mg/kg Cyclo; ALL-50 received 0.25 mg/kg VCR and 70 mg/kg Cyclo; ALL-265 received 0.3 mg/kg VCR and 70 mg/kg Cyclo. In the ALL-199 "low dose" treatment course 0.1 mg/kg VCR and 50 mg/kg Cyclo was used, in the short term treatment scheme, mice bearing ALL-199 or ALL-265 received 0.5 mg/kg VCR and 100 mg/kg Cyclo and in the "high dose" treatment scheme, mice received 0.6 mg/kg VCR and 100 mg/kg Cyclo. Mice were closely monitored to prevent signs of treatment toxicity and sacrificed as soon as clinical signs of sickness became apparent (weight loss, rough fur, reduced motility, hunchback).

Criteria to define sensitive, persisting and resistant cells were as follows: *Sensitive:* imaging values in the "descending phase" in repetitive imaging, 2-3 weeks after start of treatment. *Persisting:* imaging values "stable" in repetitive imaging at or below 1% relative to start of treatment. *Resistant:* imaging values in the "ascending phase" in repetitive imaging; values rise by at least 10x compared to tumor burden at the persisting stage, despite continuous treatment.

Drug holiday experiments: resistant ALL-199 derivatives were injected into mice (n=1 per derivative) and grown without treatment. At BLI signals ≥ $1x10^{10}$ p/s, PDX cells were re-isolated from murine BM and up to $5x10^6$ cells were re-transplanted into next recipient mice. The procedure was repeated twice, resulting in approximately 6 month

of drug holiday. In the fourth passage, cells were injected into two mice per derivative. One mouse was left untreated while the other mouse received chemotherapy, starting at a BLI signal ≥ $1x10^9$ p/s. Treatment response was analyzed by repetitive BLI.

Pretreatment experiments: mice were treated with chemotherapy or with PBS as a control for six weeks, before donor ALL-199 PDX cells were injected. ALL-199 cells were either injected in low numbers (n=5 mice per group) to analyze tumor growth by repetitive BLI and compare growth between pre-treated and untreated mice, or in high cell numbers to achieve a high tumor burden (n=5 mice per group) and initiate treatment with combination chemotherapy; treatment response was monitored by repetitive BLI.

Multiplexing PDX

For the multiplex in vivo experiment, individual PDX samples were selected to i) be suitable for transduction and in vitro cultivation; ii) cover a range of different ALL subtypes (Ph-like, hyperdiploid, TCF3-TBX1, KMT2A-AFF1, DUX4 fusion); iii) cover different stages of disease (initial diagnosis, first and second relapse); and iv) have a similar passaging time in vivo. PDX cells were marked by one or more fluorochromes (eGFP, mCherry, mtagBFP, iRFP or combinations of these) as described (1, 8). Transduced cells were kept in vitro for 3 days, followed by enrichment by FACS. A mix of all samples was injected into two mice to expand the transgenic cell populations. At high leukemic burden, cells were re-isolated from mice and individual populations were enriched by FACS. Fluorochrome expression profiles of each sample were recorded by flow cytometry and samples were mixed again. Resistant PDX ALL-199 cells, which were known to proliferate slower than untreated PDX ALL-199, were injected in a larger proportion (10x) compared to untreated PDX ALL-199. For ALL-50 and ALL-707, fewer cells had to be injected as less cells could be isolated from donor mice. Individual samples were injected in the ratio: 1x ALL-199U, 10x ALL-199R, 0.07x ALL-50, 1x ALL-265, 1x ALL-502, 0,75x ALL-707. Fluorochrome profiles of the mix were recorded as well and cells were injected into groups of mice (n=12). Engraftment was analyzed by repetitive BLI. At BLI signal ≥ $1x10^9$ p/s, mice were randomly assigned to one of three groups (n=4 per group). One group was sacrificed, PDX cells were re-isolated from murine bone marrow and composition of the PDX population was analyzed by flow cytometry. One group received polychemotherapy for three weeks, one group

received PBS as control. At the end of the experiment, PDX cells were re-isolated from murine bone marrow and fluorochrome expression analyzed to identify individual PDX populations. Relative proportion of the individual PDX sample within the mixture was determined for each PDX sample in each group. Proportion after treatment and in the PBS treated group was normalized to start of treatment.

Determination of doubling time of PDX in vivo

In vivo doubling time was calculated by non-linear fitting of an exponential growth curve to data points of repetitive *in vivo* imaging using GraphPad prism. Doubling time was based on two different time points (($x_1|y_1$) and ($x_2|y_2$)) and calculated as follows: Doubling time = ln(2)/k with $k = (\ln(y_2)-\ln(y_1))/(x_2-x_1)$.

Exome Sequencing and data analysis

DNA isolation and exome sequencing was performed at LaFuGa (Gene Center, LMU Munich). Murine bone marrow containing PDX-ALL cells was thawed and human cells were enriched. gDNA and library preparation was performed using QIAmp DNA Blood Mini Kit (Qiagen, Düren, Germany) and quantification by Nanodrop and Qubit dsDNA HS Assay Kit. Exome libraries were prepared and multiplexed using SureSelect Human All Exon V6 kit. Sequencing was performed using Illumina HiSeq 1500 and NextSeq 2000 systems with 100 bp or 150 bp paired-end reads. For data analysis and quality assessment, sequencing reads with original length of 150bp were trimmed to a length of 100bp at the 5' end to ensure consistent mapping. All sequences were trimmed based on base calling quality (min 13) at both ends. Sequence reads containing "N" bases and reads with length smaller than 50 nucleotides after trimming were discarded. Reads were mapped to the hg19 reference genome using BWA-aln 0.7.10 with default parameters. Mapped reads were filtered based on mapping quality (min 13) and reads not mapping to annotated protein coding regions were discarded. Reads likely arising from PCR duplicates were removed using samtools rmdup. On average, about 43 million high quality reads per sample were used in the analysis (range 29,575,575 - 58,527,169), resulting in an average target coverage of 80x. Sequencing reads were realigned around insertions and deletions using GATK IndelRealigner. Sequence variants in individual samples were detected using samtools mpileup and VarScan 2.3.7 using a minimum variant allele frequency (VAF) of 20%

with a minimum coverage of 10x and the number of variant supporting reads ≥3, and a base calling quality of 20. Low evidence variants found in donor sample were subtracted to call variants gained in all other samples. Low evidence variants in donor sample were called using reduced cutoffs: VAF ≥5%, coverage ≥1x, number of variant supporting reads ≥1, base calling quality ≥6. The translational effect of sequence variants was annotated using SnpEff 4.3 and the Ensembl GRCh37.75 annotation. Copy number alterations were detected using GATK DepthOfCoverage and OptimalCaptureSegmentation as previously described (9). The following cutoffs were applied: mean exon coverage ≥10x, number of exons per segment ≥2, size of segments ≥100kb, and number of segments per chromosome ≤10. Four samples were excluded from further analyses due to poor sequencing quality (ALL-199 D3, ALL-50 D6, and two ALL-199 untreated control samples). VAF were filtered based on their genomic position and frequency. Mutations in regions of copy number loss were extracted by filtering for the respective genomic regions Protein-coding
genes assigned to these genomic regions were extracted using Ensembl Biomart with genome assembly GRCh37.p13 (10).

Targeted sequencing of recurrently mutated genes

To obtain a high sequencing depth of identified *TP53* mutations in ALL-199, an established sequencing panel of 68 recurrently mutated genes was utilized. Experimental procedure and data analysis was performed as previously described (11).

Transcriptome sequencing with prime-seq and differential gene expression analysis

Murine bone marrow containing PDX-ALL was thawed and 2000 human cells were enriched by FACS based on GFP expression. Cells were sorted into 100 µL RLT Plus Buffer (Qiagen, Düren, Germany) supplemented with 1 % β-Mercaptoethanol. Samples were flash frozen and stored at -80 °C until further processing. RNA sequencing was performed using prime-seq (12), a bulk version of the single cell RNA-seq method mcSCRB-seq (13). In brief, samples were digested with Proteinase K and nucleic acids were isolated using SPRI Beads and DNAseI digestion was performed on beads. Reverse transcription of the isolated RNA was done using barcoded oligo-dT primers and a template switching oligo. Exonuclease I digestion was performed to

remove excess primers. cDNA was amplified using Kapa HiFi HotStart polymerase and quality was assessed using capillary gel electrophoresis. Library preparation was carried out in triplicates with 0.8 ng cDNA input each using the Nextera XT Kit (Illumina, San Diego, USA). Fragments of 300-900 bp size were selected by agarose gel electrophoresis and library sequencing was performed at LaFuGA (LMU, Munich) with Illumina HiSeq1500 (Illumina, San Diego, USA) with paired-end reads. Sequencing was designed to cover barcode and UMI sequences with 28 bp in the first read and cDNA fragments with 50 bp in the second read and aiming for at least 10 Mio reads per sample. Raw fastq files were processed using the zUMIs pipeline (14). Reads were mapped to a concatenated genome of human and mouse (hg38, mm10) and Ensembl gene models (GRCh38 v.84,GRCm38.85) were used for quantification of gene expression levels. Samples were analyzed in two different batches according to the same protocol with minor modifications. The first batch was carried out using individual amplification of cDNAs, the second batch using pooled amplification for less bias and improved quality. To correct the batch effect resulting from the use of different prime-seq sequencing time points, an empirical Bayesian method or the methodologies implemented in the limma package of R were applied as described previously (15, 16). Normalization of read counts was performed using the voom function (17). Differential gene expression was calculated using the DESeq2 and limma packages in R following recommended workflows (16, 17). Differential gene expression was calculated between resistant and untreated samples and significantly upregulated transcripts in resistant samples were defined with the following cut-offs: $p < 0.001$ and $\log_2$ fold-change >1.

Differential gene expression analysis (t-test) between 1p-deleted (n=15; derivatives D4-D8) vs. 1p non-deleted (n=10, derivatives D1-D2) resistant derivatives and corresponding data visualization were done using the R2: Genomics Analysis and Visualization Platform (http://r2.amc.nl). *BCL2* expression in high risk B-precursor ALL samples was analyzed from the R2 deposited dataset "Tumor ALL (B) - Carroll - 98 - MAS5.0 - u133p2" (based on GSE7440) (18).

To test the association between *BCL2* and overall survival, we used the publicly available microarray data set consisting of 449 AML patients treated in different trials of the Haemato-Oncology Foundation for Adults in the Netherlands (HOVON). The data were a part of a publicly available cohort analyzed by Affymetrix (GSE14468) (19, 20).

Protein expression analysis

Proteome analysis was performed as described previously (21). Murine bone marrow containing PDX ALL was thawed and human cells were enriched by FACS based on GFP expression and washed thoroughly in PBS twice. Cells were lysed in 1% SDC buffer, incubated (15 min, on ice), boiled (5 min, 95 °C), sonicated for 20 cycles and boiled (5 min, 95 °C). Proteins were digested with LysC (1:100 ratio) and Trypsin (1:100 ratio) for 16 hours at 37°C and reaction was stopped using 5:1 2-propanol/1% TFA. Resulting peptides were de-salted on equilibrated styrenedivinylbenzene-reversed phase sulfonated (SDB-RPS) StageTips, washed once in isopropanol/1% TFA and twice with 0.2% TFA, eluted with 60 µl elution buffer (80% ACN, 1.25% NH_4OH) and dried under vacuum. The dried peptides were resuspended in loading buffer (3%ACN, 0.3% TFA) and subjected to mass spectrometry (MS).

MS analysis was performed using Q Exactive HF-X Hybrid Quadrupole-Orbitrap Mass Spectrometer (Thermo Fisher Scientific, Waltham, USA) coupled to a nanoflow EASY-nLC1000 HPLC (Thermo Fisher Scientific, Waltham, USA). App. 500 ng peptide was loaded onto a 50 cm column with 75 µM diameter, packed in house with 1.9 µM C18 ReproSil particles, and column temperature was maintained at 50 °C (Dr. Maisch, Ammerbuch, Germany). Peptides were separated using a gradient of a two-buffer system of 0.1% formic acid and 60% ACN plus 0.1%formic acid for 140 min at a flow rate of 300 nl/min. The survey scans were acquired at a resolution of 60,000 FWHM at a AGC target of 3 x 10^6 ions (300-1650 m/z, 200 m/z, maximum filling time 20ms), followed by HCD (high energy collisional dissociation) fragmentation of Top15 dynamically chosen ions. The MS/MS scans were detected in the Orbitrap at a resolution of 15,000 FWHM. Data analysis was performed using Maxquant version 1.5.5.2 (22). The MS/MS spectra were searched for tryptic peptides using a target-decoy approach with a reverse database from the reference Uniprot Human (version 2016) proteome and list of potential contaminants by the build in Andromeda search engine. Settings were adjusted as follows: max. of two missed cleavages allowed, minimum peptide length of seven amino acids, Carbamidomethyl of cysteine (C) as fixed modification, oxidized methionine (M), acetylation (protein N-term) as variable modification, false discovery rate of less than 1% at the levels of peptide and protein identification. The proteins were assigned to the same protein groups if two proteins could not be discriminated by unique peptides. The label-free quantification was

performed with the MaxLFQ algorithum (23) requiring a minimum ratio count of two. Match between run feature was enabled for identification of peptide across runs based on mass accuracy and normalized retention times. After filtering of artefacts, such as reverse hits and contaminants, Perseus software was used to analyze Maxquant output tables (24).

Gene set enrichment analysis

Gene set enrichment analysis was performed using GSEA Desktop Application Version 4.2.3. (25, 26). Expression datasets of resistant and untreated PDX ALL-199 cells of transcriptome and proteome analysis were submitted to GSEA using 1000 permutations, Chip platform Human_ENSEMBL_Gene_ID_MSigDB.v7.5.1 and gene sets Hallmarks (h.all.v7.5.1.symbols.gmt), KEGG (c2.cp.kegg.v7.5.1.symbols.gmt) and GO Terms (c5.go.v7.5.1.symbols.gmt) and analyzed for gene sets enriched in resistant samples.

CRISPR/Cas9 dropout screens in ALL PDX samples

CRISPR-Cas9 dropout screens were performed using a protocol that we had optimized to be performed in vivo in PDX models (27, 28). Cloning of humanized Streptococcus pyogenes (hSp) Cas9 into lentiviral vectors was previously described (5). Fluorochrome marker was changed to mCherry using restriction cloning and cutting sites for NotI and SalI. Cloning of sgRNA libraries was performed as previously described (28), except that a H2Kk-mtagBFP marker was used to allow both magnetic enrichment and FACS enrichment. The CLUE pipeline was used to generate a library of 1196 sgRNAs, including sgRNAs targeting candidate genes, non-targeting negative controls and sgRNAs targeting essential genes as positive controls (28-30), Resistant ALL-199 cells were first transduced with the Cas9 construct and cultured for 3 days in vitro before cells were enriched by FACS sorting for mCherry and injected into one mouse per resistant derivative. At high tumor burden, cells were re-isolated from murine bone marrow and percentage of Cas9-positive cells was assessed by flow cytometry. Cas9/mCherry positive cells were enriched by FACS sorting and this procedure was repeated twice to generate a pure population of Cas9-positive PDX cells. Cas9-positive cells were injected into groups of mice (n=5-6) for expansion. At high tumor burden, cells were re-isolated from murine bone marrow and transduced with the sgRNA library, aiming for a maximal transduction efficiency of 30% to achieve

single integrations of sgRNAs. Cells were cultured for 3 days in vitro before transduction efficiency was analyzed by flow cytometry and transduced cells were magnetically enriched using the H2Kk-marker. Cas9- and sgRNA-library double-positive cells were injected into groups of mice, aiming for at least 100x coverage per sgRNA within the homed population, i.e. at least $2.4x10^6$ cells were injected assuming a homing frequency of 5% with a library size of 1196 sgRNAs. One fraction was used as input control. For the screen using D5, n=11 mice were injected with $2.4x10^6$ cells per mouse. For the screen using D7, n=11 mice were injected with $6x10^6$ cells per mouse. At BLI signal ≥ $1x10^9$ P/s, mice were randomly distributed and either sacrificed (treatment start) or treated with polychemotherapy (treated) or solvent (PBS). After three weeks, cells were re-isolated from murine bone marrow and samples were subjected to NGS as previously described (28). Significant dropouts were identified using the model-based analysis of genome-wide CRISPR/Cas9 knockout (MAGeCK)(31) using the MAGeCK pipeline based on the Galaxy server (32). To analyze the evenness of sgRNA read counts in the library plasmid pool, fraction of sgRNAs was plotted against fraction of total read counts and GINI Index was calculated (33).

Barcoding of PDX cells

Barcodes were cloned and PDX cells transduced as previously described (8). For barcode analysis, gDNA was isolated from the pelleted, flash-frozen ALL PDX cells using the DNeasy Blood and Tissue Kit (Qiagen). When the total cell number exceeded $5*10^6$ cells, the sample was split into two columns and combined after elution. Based on the fraction of human cells as measured by Flow Cytometry, the total number of human genomes per 5 µl of gDNA was calculated and gDNA was concentrated using a SPRI bead clean-up to achieve more than 1000 genomes per sample to ensure comprehensive barcode detection. The Library amplification protocol is based on SiMSen-Seq (34, 35) consisting of a barcoding PCR and an adapter PCR; the first PCR adds unique molecular identifiers (UMI) in a limited number of cycles to ensure uniqueness of the UMI's, the second PCR adds the necessary sequencing adapters and sample indices needed for Illumina sequencing. To ensure recovery of the full barcode complexity in the gDNA, the reaction was performed in triplicates. Due to the low on-target ratios in some samples the barcoding PCR was performed for 6 cycles. Based on the results of a PCR cycling test, samples were amplified for 20 - 28 cycles

to a final concentration between 1 and 5 ng/µL. Each Replicate was indexed with a unique i5 index and an i7 index that was shared between all replicates of a sample. Library quantification was performed using picogreen (Thermo Fisher) and all samples were pooled equimolar. The final library was quantified and correct amplicon size was confirmed using capillary gel electrophoresis (Agilent, Bioanalyzer DNA HS). Barcode sequencing was performed on an Illumina HiSeq 1500 instrument for 110 cycles covering the barcode and UMI sequences. Raw fastq files were demultiplexed using deML (https://github.com/grenaud/deML). Barcode assignment and clustering of barcodes to account for sequencing errors and polymerase errors was performed using bartender (36). Clusters with a distance of less than or equal to 4 were collapsed. All further processing and visualisation was performed in R version 3.6. To account for differences in sequencing depth counts were transformed to cpm. Replicates were combined by taking the average cpm value per barcode.

Statistics

All statistical analyses were performed using GraphPad Prism 7.05 or R 3.6.1. Statistical testing between two groups was performed using unpaired t-test. Comparison between more than two groups was calculated with one-way ANOVA followed by Tukey´s multiple comparison test. Levels of significance were defined as follows: $p > 0.05$: n.s., $p \leq 0.05$: *, $p \leq 0.01$: **, $p \leq 0.001$: ***. Correlation of flow cytometry and bioluminescence *in vivo* imaging signal intensity was calculated using non-linear regression.

Supplemental References

1. Carlet M, Volse K, Vergalli J, Becker M, Herold T, Arner A, et al. In vivo inducible reverse genetics in patients' tumors to identify individual therapeutic targets. Nat Commun. 2021 Sep 27;12(1):5655. Epub 2021/09/29. doi:10.1038/s41467-021-25963-z. Cited in: Pubmed; PMID 34580292.

2. Ebinger S, Ozdemir EZ, Ziegenhain C, Tiedt S, Castro Alves C, Grunert M, et al. Characterization of Rare, Dormant, and Therapy-Resistant Cells in Acute Lymphoblastic Leukemia. Cancer Cell. 2016 Dec 12;30(6):849-862. Epub 2016/12/06. doi:10.1016/j.ccell.2016.11.002. Cited in: Pubmed; PMID 27916615.

3. Terziyska N, Castro Alves C, Groiss V, Schneider K, Farkasova K, Ogris M, et al. In vivo imaging enables high resolution preclinical trials on patients' leukemia cells growing in mice. PLoS One. 2012;7(12):e52798. eng. Epub 2013/01/10. doi:10.1371/journal.pone.0052798. Cited in: Pubmed; PMID 23300782.

4. Heckl BC, Carlet M, Vick B, Roolf C, Alsadeq A, Grunert M, et al. Frequent and reliable engraftment of certain adult primary acute lymphoblastic leukemias in mice. Leuk Lymphoma. 2019 Mar;60(3):848-851. eng. Epub 2018/09/21. doi:10.1080/10428194.2018.1509314. Cited in: Pubmed; PMID 30234406.

5. Liu WH, Mrozek-Gorska P, Wirth AK, Herold T, Schwarzkopf L, Pich D, et al. Inducible transgene expression in PDX models in vivo identifies KLF4 as a therapeutic target for B-ALL. Biomarker research. 2020;8:46. eng. Epub 2020/09/19. doi:10.1186/s40364-020-00226-z. Cited in: Pubmed; PMID 32944247.

6. Sharma V, McNeill JH. To scale or not to scale: the principles of dose extrapolation. Br J Pharmacol. 2009 Jul;157(6):907-21. Epub 2009/06/11. doi:10.1111/j.1476-5381.2009.00267.x. Cited in: Pubmed; PMID 19508398.

7. Nair AB, Jacob S. A simple practice guide for dose conversion between animals and human. J Basic Clin Pharm. 2016 Mar;7(2):27-31. Epub 2016/04/09. doi:10.4103/0976-0105.177703. Cited in: Pubmed; PMID 27057123.

8. Zeller C, Richter D, Jurinovic V, Valtierra-Gutiérrez IA, Jayavelu AK, Mann M, et al. Adverse stem cell clones within a single patient’s tumor predict clinical outcome in AML patients. Journal of Hematology & Oncology. 2022 2022/03/12;15(1):25. doi:10.1186/s13045-022-01232-4.

9. Vosberg S, Herold T, Hartmann L, Neumann M, Opatz S, Metzeler KH, et al. Close correlation of copy number aberrations detected by next-generation sequencing with results from routine cytogenetics in acute myeloid leukemia. Genes Chromosomes Cancer. 2016 Jul;55(7):553-67. Epub 2016/03/26. doi:10.1002/gcc.22359. Cited in: Pubmed; PMID 27015608.

10. Yates AD, Achuthan P, Akanni W, Allen J, Allen J, Alvarez-Jarreta J, et al. Ensembl 2020. Nucleic Acids Res. 2020 Jan 8;48(D1):D682-D688. Epub 2019/11/07. doi:10.1093/nar/gkz966. Cited in: Pubmed; PMID 31691826.

11. Metzeler KH, Herold T, Rothenberg-Thurley M, Amler S, Sauerland MC, Gorlich D, et al. Spectrum and prognostic relevance of driver gene mutations in acute myeloid leukemia. Blood. 2016 Aug 4;128(5):686-98. Epub 2016/06/12. doi:10.1182/blood-2016-01-693879. Cited in: Pubmed; PMID 27288520.

12. Janjic A, Wange LE, Bagnoli JW, Geuder J, Nguyen P, Richter D, et al. Prime-seq, efficient and powerful bulk RNA-sequencing. bioRxiv. 2021:2021.09.27.459575. doi:10.1101/2021.09.27.459575.

13. Bagnoli JW, Ziegenhain C, Janjic A, Wange LE, Vieth B, Parekh S, et al. Sensitive and powerful single-cell RNA sequencing using mcSCRB-seq. Nat Commun. 2018 Jul 26;9(1):2937. Epub 2018/07/28. doi:10.1038/s41467-018-05347-6. Cited in: Pubmed; PMID 30050112.

14. Parekh S, Ziegenhain C, Vieth B, Enard W, Hellmann I. zUMIs - A fast and flexible pipeline to process RNA sequencing data with UMIs. Gigascience. 2018 Jun 1;7(6). Epub 2018/05/31. doi:10.1093/gigascience/giy059. Cited in: Pubmed; PMID 29846586.

15. Johnson WE, Li C, Rabinovic A. Adjusting batch effects in microarray expression data using empirical Bayes methods. Biostatistics. 2007 Jan;8(1):118-27. Epub 2006/04/25. doi:10.1093/biostatistics/kxj037. Cited in: Pubmed; PMID 16632515.

16. Ritchie ME, Phipson B, Wu D, Hu Y, Law CW, Shi W, et al. limma powers differential expression analyses for RNA-sequencing and microarray studies. Nucleic Acids Res. 2015 Apr 20;43(7):e47. Epub 2015/01/22. doi:10.1093/nar/gkv007. Cited in: Pubmed; PMID 25605792.

17. Love MI, Huber W, Anders S. Moderated estimation of fold change and dispersion for RNA-seq data with DESeq2. Genome Biol. 2014;15(12):550. Epub 2014/12/18. doi:10.1186/s13059-014-0550-8. Cited in: Pubmed; PMID 25516281.

18. Bhojwani D, Kang H, Menezes RX, Yang W, Sather H, Moskowitz NP, et al. Gene expression signatures predictive of early response and outcome in high-risk childhood acute lymphoblastic leukemia: A Children's Oncology Group Study [corrected]. J Clin Oncol. 2008 Sep 20;26(27):4376-84. eng. doi:10.1200/jco.2007.14.4519. Cited in: Pubmed; PMID 18802149.

19. Wouters BJ, Löwenberg B, Erpelinck-Verschueren CAJ, van Putten WLJ, Valk PJM, Delwel R. Double CEBPA mutations, but not single CEBPA mutations, define a subgroup of acute myeloid leukemia with a distinctive gene expression profile that is uniquely associated with a favorable outcome. Blood. 2009;113(13):3088-3091. eng. Epub 2009/01/26. doi:10.1182/blood-2008-09-179895. Cited in: Pubmed; PMID 19171880.

20. Taskesen E, Bullinger L, Corbacioglu A, Sanders MA, Erpelinck CA, Wouters BJ, et al. Prognostic impact, concurrent genetic mutations, and gene expression features of AML with CEBPA mutations in a cohort of 1182 cytogenetically normal AML patients: further evidence for CEBPA double mutant AML as a distinctive disease entity. Blood. 2011 Feb 24;117(8):2469-75. eng. Epub 20101221. doi:10.1182/blood-2010-09-307280. Cited in: Pubmed; PMID 21177436.

21. Kulak NA, Pichler G, Paron I, Nagaraj N, Mann M. Minimal, encapsulated proteomic-sample processing applied to copy-number estimation in eukaryotic cells. Nat Methods. 2014 Mar;11(3):319-24. Epub 2014/02/04. doi:10.1038/nmeth.2834. Cited in: Pubmed; PMID 24487582.

22. Cox J, Mann M. MaxQuant enables high peptide identification rates, individualized p.p.b.-range mass accuracies and proteome-wide protein quantification. Nat Biotechnol. 2008 Dec;26(12):1367-72. Epub 2008/11/26. doi:10.1038/nbt.1511. Cited in: Pubmed; PMID 19029910.

23. Cox J, Hein MY, Luber CA, Paron I, Nagaraj N, Mann M. Accurate proteome-wide label-free quantification by delayed normalization and maximal peptide ratio extraction, termed MaxLFQ. Mol Cell Proteomics. 2014 Sep;13(9):2513-26. Epub 2014/06/20. doi:10.1074/mcp.M113.031591. Cited in: Pubmed; PMID 24942700.

24. Tyanova S, Temu T, Sinitcyn P, Carlson A, Hein MY, Geiger T, et al. The Perseus computational platform for comprehensive analysis of (prote)omics data. Nat Methods.

2016 Sep;13(9):731-40. Epub 2016/06/28. doi:10.1038/nmeth.3901. Cited in: Pubmed; PMID 27348712.

25. Subramanian A, Tamayo P, Mootha VK, Mukherjee S, Ebert BL, Gillette MA, et al. Gene set enrichment analysis: a knowledge-based approach for interpreting genome-wide expression profiles. Proc Natl Acad Sci U S A. 2005 Oct 25;102(43):15545-50. eng. Epub 20050930. doi:10.1073/pnas.0506580102. Cited in: Pubmed; PMID 16199517.

26. Mootha VK, Lindgren CM, Eriksson KF, Subramanian A, Sihag S, Lehar J, et al. PGC-1alpha-responsive genes involved in oxidative phosphorylation are coordinately downregulated in human diabetes. Nat Genet. 2003 Jul;34(3):267-73. eng. doi:10.1038/ng1180. Cited in: Pubmed; PMID 12808457.

27. Bahrami E, Becker M, Wirth A-K, Schmid JP, Herold T, Öllinger R, et al. A CRISPR/Cas9 Library Screen in Patients' Leukemia Cells In Vivo. Blood. 2019;134(Supplement_1):3945-3945. doi:10.1182/blood-2019-124288.

28. Becker M, Noll-Puchta H, Amend D, Nolte F, Fuchs C, Jeremias I, et al. CLUE: a bioinformatic and wet-lab pipeline for multiplexed cloning of custom sgRNA libraries. Nucleic Acids Res. 2020 Jul 27;48(13):e78. Epub 2020/06/02. doi:10.1093/nar/gkaa459. Cited in: Pubmed; PMID 32479629.

29. Doench JG, Fusi N, Sullender M, Hegde M, Vaimberg EW, Donovan KF, et al. Optimized sgRNA design to maximize activity and minimize off-target effects of CRISPR-Cas9. Nature Biotechnology. 2016 2016/02/01;34(2):184-191. doi:10.1038/nbt.3437.

30. Sanson KR, Hanna RE, Hegde M, Donovan KF, Strand C, Sullender ME, et al. Optimized libraries for CRISPR-Cas9 genetic screens with multiple modalities. Nature Communications. 2018 2018/12/21;9(1):5416. doi:10.1038/s41467-018-07901-8.

31. Li W, Xu H, Xiao T, Cong L, Love MI, Zhang F, et al. MAGeCK enables robust identification of essential genes from genome-scale CRISPR/Cas9 knockout screens. Genome Biol. 2014;15(12):554. Epub 2014/12/06. doi:10.1186/s13059-014-0554-4. Cited in: Pubmed; PMID 25476604.

32. Afgan E, Baker D, Batut B, van den Beek M, Bouvier D, Cech M, et al. The Galaxy platform for accessible, reproducible and collaborative biomedical analyses: 2018

update. Nucleic Acids Res. 2018 Jul 2;46(W1):W537-W544. Epub 2018/05/24. doi:10.1093/nar/gky379. Cited in: Pubmed; PMID 29790989.

33. Wang B, Wang M, Zhang W, Xiao T, Chen CH, Wu A, et al. Integrative analysis of pooled CRISPR genetic screens using MAGeCKFlute. Nat Protoc. 2019 Mar;14(3):756-780. Epub 2019/02/03. doi:10.1038/s41596-018-0113-7. Cited in: Pubmed; PMID 30710114.

34. Ståhlberg A, Krzyzanowski PM, Jackson JB, Egyud M, Stein L, Godfrey TE. Simple, multiplexed, PCR-based barcoding of DNA enables sensitive mutation detection in liquid biopsies using sequencing. Nucleic Acids Res. 2016 Jun 20;44(11):e105. eng. Epub 2016/04/10. doi:10.1093/nar/gkw224. Cited in: Pubmed; PMID 27060140.

35. Ståhlberg A, Krzyzanowski PM, Egyud M, Filges S, Stein L, Godfrey TE. Simple multiplexed PCR-based barcoding of DNA for ultrasensitive mutation detection by next-generation sequencing. Nat Protoc. 2017 Apr;12(4):664-682. eng. Epub 2017/03/03. doi:10.1038/nprot.2017.006. Cited in: Pubmed; PMID 28253235.

36. Zhao L, Liu Z, Levy SF, Wu S. Bartender: a fast and accurate clustering algorithm to count barcode reads. Bioinformatics (Oxford, England). 2018 Mar 1;34(5):739-747. eng. Epub 2017/10/27. doi:10.1093/bioinformatics/btx655. Cited in: Pubmed; PMID 29069318.

Supplemental Table S1. Clinical characteristics of ALL patients donating cells

sample	disease stage*	age [years]	sex	cytogenetics and gene fusions	mutations∞	Reference
**ALL-50**	ID	7	F	TCF3-PBX1; t(1;19)	N.D.	[1,2]
**ALL-199**	R2	8	F	germline trisomy 21; Somatic: homozygous 9p deletion (CDKN2A); P2RY8-CRLF2 fusion (Ph-like subtype)	KRAS	[1,2,3,4,5]
**ALL-265**	R1	5	F	hyperdipoidy (+6,+13,+14,+17,+18,+21,+X)	KMT2D, HERC1, CSMD1, PRRT2	[1,3,4,5]
**ALL-502**	R	10	F	DUX4 fusion	N.D.	
**ALL-707**	ID	2	M	t(4;11) KMT2A-AFF1	N.D.	[1,4]

*when the primary sample was obtained; ∞ mutations determined by panel sequencing;
ID = initial diagnosis; R1 = 1st relapse; R2 = 2nd relapse; F = female; M = male; N.D. not determined.

[1] Heckl et al., Leuk Lymphoma 2019
[2] Terziyska et al., PlosOne 2012
[3] Ebinger et al., Cancer Cell 2016
[4] Carlet et al., Nat Comm 2021
[5] Liu et al., Biomark Res 2020

Supplemental Table S2. Somatic CNAs in PDX ALL-199 donor and resistant derivatives
Start and end position of hg19 reference genome. Ratio was calculated as sample/complete remission. Only ratio >1.1 or <0.9 are listed.

Sample	Chr	Start	Stop	NumExons	Size(bp)	Ratio
Donor	1	219352484	249212567	2050	29860083	0.4991
Donor	6	25921229	33385092	1668	7463863	1.2423
Donor	6	117639346	170893674	2647	53254328	1.4260
Donor	7	48685008	55606355	130	6921347	0.4750
Donor	13	41556114	115091761	2235	73534527	1.4086
Donor	14	19377589	25326368	1118	5939469	1.9145
Donor	17	25636121	39767487	2269	14121763	1.5241
Donor	17	39767592	81052325	5776	41284733	1.5642
Donor	19	6833190	15383915	2247	8550725	1.6591
Untreated	1	220088786	249212567	2046	29123781	0.5068
Untreated	6	117252489	170893674	2658	53641185	1.4850
Untreated	7	48685008	56140809	179	7455801	0.5669
Untreated	7	56141862	65444903	83	9303041	1.4464
Untreated	13	41556114	78218414	1096	36659525	1.4553
Untreated	13	78272044	115091761	1139	36819717	1.4752
Untreated	14	19377589	24518025	731	5133266	1.8362
Untreated	17	25638530	39767487	2267	14119354	1.4480
Untreated	17	39767592	81052325	5776	41284733	1.4886
Untreated	19	6833190	15383915	2247	8550725	1.5561
D1	1	159141551	169799487	1009	10657434	1.5453
D1	1	169799832	218614709	3348	48814877	1.4602
D1	1	219347228	249212567	2051	29865339	0.4993
D1	6	25921229	33382326	1659	7461097	1.2557
D1	6	117641026	148846506	1375	31205480	1.7829
D1	6	148848584	170893674	1271	22045090	1.9847
D1	7	48685008	55606355	130	6921347	0.4641
D1	13	41556114	115091761	2235	73528154	1.4044
D1	14	19377589	24518025	731	5137873	1.8394
D1	17	6006	25637205	3515	25631199	0.5485
D1	19	7075661	15383915	2205	8308254	1.6969
D1	X	109693841	134978493	1001	25270278	0.5904
D1	X	134983713	155240079	1167	20256366	0.6029
D2	1	219347228	249212567	2051	29865339	0.5114
D2	6	117638301	170893674	2648	53255373	1.4762
D2	7	48685008	56130783	176	7445775	0.5607
D2	7	56131943	65445401	87	9313458	1.4955
D2	13	41639147	115091761	2232	73398113	1.3384
D2	14	19377589	24518025	731	5135702	1.7665
D2	17	6006	25636303	3514	25630297	0.5546
D2	19	6833190	15383915	2247	8550725	1.5696
D4	1	69085	31740784	4135	31671699	0.5197
D4	1	219347228	249212567	2051	29865339	0.5110
D4	6	117638301	170893674	2648	53255373	1.5261
D4	7	48685008	56140809	179	7455801	0.5872
D4	7	56141862	65444903	83	9303041	1.4762
D4	13	41639147	96684230	1309	55041881	1.4888
D4	13	96705404	115091761	923	18386357	1.4668
D4	17	26091002	39594873	2163	13494353	1.4045
D4	17	39594944	81052325	5843	41457381	1.4430
D4	19	6833190	24310787	3582	17,334,207	1.5033
D5	1	69085	28537153	3939	28468068	0.5373
D5	1	156518135	218614709	4804	62096574	1.4725
D5	1	219347228	249212567	2051	29865339	0.5114
D5	6	117252489	149959688	1448	32707199	1.8339
D5	6	149982860	170893674	1210	20910814	2.0160
D5	7	48685008	56144587	182	7459579	0.5824
D5	7	56145790	65444903	80	9299113	1.5071
D5	13	41556114	115091761	2235	73528008	1.4219
D5	14	19377589	24518025	731	5137873	1.8630
D5	17	25638530	39780766	2278	14132633	1.4524
D5	17	39845286	81052325	5765	41207039	1.5162
D5	19	6833190	15383915	2247	8550725	1.6231
D6	1	69085	17087602	2243	16913580	0.5139
D6	1	17090904	32658337	2108	15567433	0.5485
D6	1	219347228	249212567	2051	29865339	0.4974
D6	6	25921229	33384558	1665	7463329	1.2251
D6	6	117641026	170893674	2646	53252648	1.4199
D6	7	48685008	55606355	130	6921347	0.4625
D6	13	41556114	78202149	1091	36642401	1.4199
D6	13	78208509	115091761	1144	36883252	1.4022
D6	14	19377589	24518025	731	5136347	1.7654
D6	17	25637082	39780766	2279	14134081	1.5252
D6	17	39845286	81052325	5765	41207039	1.5719
D6	19	110674	6821875	1848	6711201	1.2959
D6	19	6822227	15383915	2258	8561688	1.7129
D7	1	69085	32043064	4169	31973979	0.5303
D7	1	149755554	161790956	2633	12035402	1.6407
D7	1	161816234	218614709	3815	56798475	1.4565
D7	1	219347228	249212567	2051	29865339	0.4990
D7	6	25921229	33391380	1673	7470151	1.2198
D7	6	117252489	149944424	1446	32691935	1.7811
D7	6	149953890	170893674	1212	20939784	1.9715
D7	7	48685008	55606355	130	6921347	0.4601
D7	7	151962118	158937468	292	6975350	0.7737
D7	13	41556114	78202149	1091	36642401	1.3984
D7	13	78208509	115091761	1144	36883252	1.4298
D7	14	19377589	24518025	731	5135702	1.7770
D7	17	25638530	39767487	2267	14119354	1.5289
D7	17	39767592	81052325	5776	41284733	1.5776
D7	19	6833190	15383915	2247	8550725	1.6930
D8	1	220088786	249212567	2046	29123781	0.4991
D8	6	25917192	33384558	1667	7467366	1.2628
D8	6	117641026	170893674	2646	53252648	1.4120
D8	7	48685008	55606355	130	6921347	0.4711
D8	7	151944982	158937468	298	6992486	0.7650
D8	13	41556114	115091761	2235	73534527	1.3973
D8	14	19377589	24518025	731	5137228	1.8605
D8	17	25638530	39777136	2273	14129003	1.5308
D8	17	39777213	81052325	5770	41275112	1.5820
D8	19	7075661	15383915	2205	8308254	1.7127

Supplemental Table S3. CNAs in PDX ALL-50 donor and resistant derivatives. Donor is compared to primary patient leukemia sample; untreated and resistant are compared to donor. Start and end position of hg19 reference genome. Ratio was calculated as sample vs. control. Only ratio >1.1 or <0.9 are listed.

Sample	Chr.	Start	Stop	NumExons	Size(bp)	Ratio
Donor	1	16903807	17086186	23	182379	1,1967
Donor	1	148016404	164790868	2842	16774464	0,8486
Donor	1	164815816	186957645	1715	22141829	0,8922
Donor	1	190067143	193219847	76	3152704	0,5816
Donor	1	196197349	227171560	2514	30974211	1,1847
Donor	1	227171790	245245469	1241	18073679	1,1391
Donor	1	245246739	249212567	207	3965828	1,2507
Donor	2	130872449	131096810	40	224361	1,1867
Donor	6	32487142	32709307	20	222165	0,5255
Donor	6	71162190	73787225	86	2625035	1,2116
Donor	6	91228972	116720834	846	25491862	1,1909
Donor	6	122775291	130414009	309	7638718	1,1803
Donor	7	193195	57529660	2924	57336465	1,3995
Donor	7	63505995	74133265	440	10627270	0,7737
Donor	7	75034460	100608374	1935	25518520	0,7677
Donor	7	100608721	102114136	194	1505415	0,7782
Donor	7	102448732	152007165	3197	49556535	0,7801
Donor	7	152008878	158937468	289	6928590	0,7605
Donor	9	14802	21481698	875	21375105	0,6312
Donor	9	21802743	27950674	125	6147931	0,0232
Donor	9	32405500	39178362	899	6772862	0,7511
Donor	9	75773447	82337955	287	6564508	0,6016
Donor	11	132306548	134257558	143	1951010	0,8946
Donor	13	92797078	95860184	69	3063106	1,1757
Donor	16	69973739	70417143	91	443404	1,1051
Donor	17	12920174	15641396	67	2721222	1,1391
Donor	21	10906900	15002226	33	3965496	0,8062
U1	1	16905683	17086990	25	181307	0,8463
U1	4	53378	337702	10	278396	1,1177
U1	5	36257038	36971143	26	714105	0,8621
U1	16	14960407	15116616	38	156209	0,8647
U1	16	20448585	20554589	20	106004	0,8007
U1	17	12913925	15642149	74	2728224	0,8519
U1	19	50542413	50666456	10	124043	1,2636
U2	1	16905683	17086990	25	181307	0,8215
U2	7	5792472	6018332	43	225860	0,8261
U2	9	140070155	140483175	194	413020	1,1122
U2	10	51620319	51829476	14	209157	0,8041
U2	11	73179445	73357676	2	178231	1,4018
U2	16	14960407	15116616	38	156209	0,8079
U2	16	20448585	20576172	29	127587	0,8109
U2	16	69996874	70182461	17	185587	0,8431
U2	17	12920383	15640842	65	2720459	0,8300
D1	1	16905683	17086772	24	181089	0,8397
D1	2	130845635	130953949	46	108314	0,8935
D1	5	148431700	148630987	22	199287	1,1731
D1	7	5940099	6042272	43	102173	0,8278
D1	10	33221438	33474657	5	253219	1,4339
D1	10	135373590	135498455	12	124865	0,8426
D1	11	134131627	134257558	46	125931	0,8858
D1	14	19377589	20002300	15	624711	0,8990
D1	16	14958437	15186476	77	228039	0,8756
D1	16	20432575	20634899	37	202324	0,8331
D1	17	13399509	15642149	66	2242640	0,8616
D1	19	21580035	22379440	30	799405	1,1215
D1	20	23546606	23966422	21	419816	0,8692
D1	22	25434722	26105936	38	671214	1,1022
D1	X	48054721	48270312	26	215591	0,8765
D2	2	132290581	132558515	6	267934	1,2348
D2	3	53922415	54615909	11	693494	0,7491
D2	9	127618514	127765854	43	147340	1,1485
D2	9	140218537	140331766	39	113229	1,2186
D2	14	31188526	31344314	7	155788	0,7072
D2	14	66975241	67346756	5	371515	1,3198
D2	21	45820137	46908363	124	1088226	1,1314
D3	1	16890433	17090980	40	200547	0,3327
D3	1	145312673	149858195	496	4545522	0,8648
D3	1	248616094	248738063	6	121969	0,3284
D3	2	97749487	98201866	124	452379	0,5977
D3	2	107021670	108507192	52	1485522	0,3956
D3	2	108604607	114709427	577	6104820	0,8688
D3	2	128947286	130953949	54	2006663	0,4634
D3	2	131975971	132290647	31	314676	0,3565
D3	3	74570204	75790892	10	1156126	0,8001
D3	4	69519750	69693271	10	173521	0,6215
D3	4	69696313	71275797	120	1579484	0,8936
D3	5	175386967	175535683	21	148716	0,1339
D3	7	66463799	66648228	12	184429	0,4479
D3	7	66660152	72733056	80	6072904	0,7914
D3	7	143880592	144071987	16	191395	0,2472
D3	7	151904380	152520605	35	616225	0,3732
D3	8	7272478	7752297	45	467684	0,8732
D3	8	144662671	145193721	244	530952	1,1143
D3	9	96098153	96278556	10	180403	0,5962
D3	10	51620319	51829476	14	209157	0,3016
D3	10	81272401	81373874	10	101473	0,4698
D3	11	49053823	55035853	39	5982030	0,6351
D3	11	88583069	89535105	49	952036	0,7593
D3	11	89536861	89645259	9	108398	0,0392
D3	13	24558028	24895911	19	324662	1,1244
D3	14	19377589	19574357	10	196768	0,0514
D3	14	19582966	20249428	14	666462	0,8002
D3	15	30669228	31203048	50	533820	0,7219
D3	15	32449804	32921008	69	471204	0,6135
D3	15	43873195	44061849	78	188654	0,6823
D3	16	14947319	15186476	82	239157	0,5046
D3	16	20448585	20576172	29	127587	0,3768
D3	16	69996874	70180136	16	183262	0,2822
D3	16	70182362	70417143	64	234781	0,8202
D3	17	13399509	15642149	66	2242640	0,5356
D3	17	18390956	18708885	44	317929	0,5059
D3	17	44407811	44632795	25	224984	0,3537
D3	18	14803728	18539894	18	3736166	0,3602
D3	19	22574313	22951207	9	376894	0,5116
D3	20	45630062	45848980	17	218918	0,8495
D3	21	10906900	15011964	35	102400	0,0167
D3	21	15013661	42830713	1078	27817052	1,2179
D3	21	42838064	48084291	859	5246227	1,2841
D3	22	16258181	16449809	11	191628	0,1214
D3	22	42524171	42970066	35	445895	0,7170
D3	X	148663883	149613883	22	950000	0,6860
D4	1	16903807	17086990	26	183183	0,7967
D4	2	97638270	98264576	131	626306	0,8943
D4	2	114195441	114513126	38	317685	0,8421
D4	3	74548805	75790892	11	1176991	0,7640
D4	5	15500781	15928615	3	427834	1,4669
D4	6	75947641	85473904	395	9526263	0,8956
D4	6	86159853	97702614	435	11542761	0,8343
D4	6	150111085	150266713	24	155628	0,8264
D4	7	66479349	72717531	77	6238182	0,8852
D4	10	21435266	22616726	56	1181460	1,1323
D4	12	7890020	8077112	27	187092	0,7879
D4	16	20448585	20634899	30	186314	0,8210
D4	17	13399509	15640842	63	2241333	0,8286
D4	20	23667720	23966616	14	298896	0,8150
D4	21	10906900	44841684	1369	29933519	1,3101
D4	21	44841849	45170431	34	328582	1,1818
D4	21	45172394	47633754	421	2460684	1,3863
D4	21	47635089	48084291	148	449202	1,3515
D4	22	24176323	24325240	40	148288	1,1122
D4	22	39175449	39353730	14	178281	1,2855
D5	1	16905683	17087602	27	181668	0,8283
D5	2	97638270	97757448	9	119178	0,7904
D5	4	109680900	109783706	23	102806	0,8399
D5	7	32914599	33044968	14	130369	0,8267
D5	7	66563523	69599562	9	3036039	0,7330
D5	9	140777191	140901341	14	124150	1,1468
D5	11	88337881	89607452	57	1269571	0,8753
D5	16	14960407	15127261	44	166854	0,8440
D5	16	20432575	20634899	37	202324	0,8213
D5	17	12913925	15640842	71	2726917	0,8654
D5	21	10906900	11098742	25	111198	0,7516

Supplemental Table S4. ***TP53*** **mutations in resistant ALL-199**

Position	Region	Mutation	Sample	VAF	# Reads
Chr17:7579699	Exon3 splice site	C>T	D2	82%	89
			Donor	0%	3204
Chr17:7577093	Exon 8, Codon R282, DNA-binding domain	insC	D1	95%	39
			Donor	0%	8233

Supplemental Table S5. Cancer-related mutations in resistant ALL-50 - Provided as Excel file

Supplemental Table S6. Expression of genes on 1p36 in 1p-deleted vs. 1p-WT resistant derivatives of ALL-199 - Provided as Excel file

Supplemental Table S7. Transcriptome of ALL-199 - Provided as Excel file

Supplemental Table S8. Proteome of ALL-199 - Provided as Excel file

Supplemental Table S9. Gene set enrichment analysis. Gene sets enriched in resistant vs. untreated ALL-199 in Transcriptome - Provided as Excel file

Supplemental Table S10. Gene set enrichment analysis. Gene sets enriched in resistant vs. untreated ALL-199 in Proteome - Provided as Excel file

Supplemental Table S11. Composition of the sgRNA library

Group	Number of genes	Number of sgRNAs
Upregulated in Proteome	81	405
Upregulated in Transcriptome	135	675
Upregulated in Proteome AND Transcriptome	7	35
Positive control	11	55
Non-targeting control	NA	26
**Total**	**234**	**1196**

Supplemental Table S12. MAGeCK analysis of CRISPR/Cas9 in vivo screen
- Provided as Excel file

Figure S1

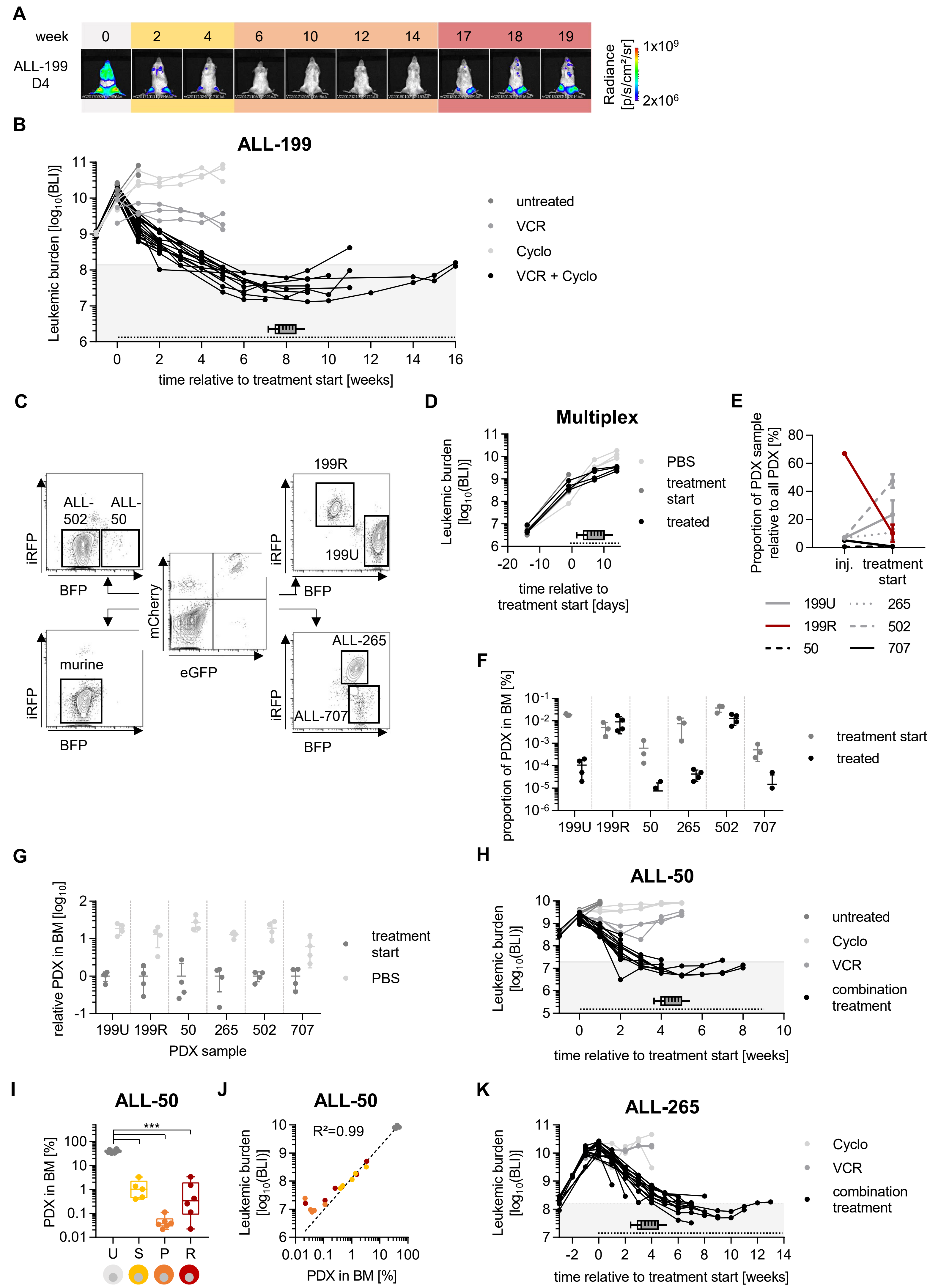

A
week
0
2
4
6
10
12
14
17
18
19
ALL-199 D4
Radiance [p/s/cm²/sr]
1x10⁹
2x10⁶
B
ALL-199
Leukemic burden [log10(BLI)]
time relative to treatment start [weeks]
untreated
VCR
Cyclo
VCR + Cyclo
C
ALL-502
ALL-50
iRFP
BFP
mCherry
eGFP
199R
199U
murine
ALL-265
ALL-707
D
Multiplex
Leukemic burden [log10(BLI)]
time relative to treatment start [days]
PBS
treatment start
treated
E
Proportion of PDX sample relative to all PDX [%]
inj.
treatment start
199U
199R
50
265
502
707
F
proportion of PDX in BM [%]
199U 199R 50 265 502 707
treatment start
treated
G
relative PDX in BM [log10]
PDX sample
treatment start
PBS
H
ALL-50
Leukemic burden [log10(BLI)]
time relative to treatment start [weeks]
untreated
Cyclo
VCR
combination treatment
I
ALL-50
PDX in BM [%]
***
U S P R
J
ALL-50
R²=0.99
Leukemic burden [log10(BLI)]
PDX in BM [%]
K
ALL-265
Leukemic burden [log10(BLI)]
time relative to treatment start [weeks]
Cyclo
VCR
combination treatment

Supplementary Figure S1. An *in vivo* model of acquired resistance in PDX ALL.
Complementary data to Figure 1

A - B PDX cells acquire resistance during prolonged treatment pressure. Complementary data to experiment depicted in Figure **1A-F**.

A Representative imaging pictures of mouse (D4) as described in Figure **1B**.

B Quantification of bioluminescence imaging (BLI) signals complementing Figure **1C.** Individual mice of single treatment groups, untreated all remaining mice not included in Figure 1C are shown.

C - G Multiplexed analysis of treatment response of 5 PDX ALL models in the same mouse.
Complementary data to experiment depicted in Figure 1**H,I**.

C Gating strategy for analysis of PDX multiplex experiment. PDX cells of 6 individual PDX populations were isolated from one representative mouse and were distinguished by specific fluorochrome marker combinations. Negative population represents murine cells.

D Quantification of bioluminescence imaging (BLI) signals of mice of the multiplex experiment described in Figure 1H. Each dot represents 1 measurement and each line represents 1 mouse (n=4 per group). Dashed line indicates treatment period.

E Proportion of each PDX sample from the experiment described in Figure 1H was measured by flow cytometry before injection (inj.) and after re-isolation from the murine bone marrow at start of treatment. Mean +/- SD of each PDX sample in 4 mice at treatment start is shown.

F Data as in Figure **1I**, except that the number of PDX cells relative to the total number of cells isolated from the murine BM is shown. One dot represents the PDX population of one PDX ALL sample within one mouse.

G Data as in Figure **1I**, except that PBS control group is shown. Proportion of each PDX sample treated with PBS was normalized to the mean proportion of the respective sample within the mix at treatment start. One dot represents the PDX population of one PDX ALL sample within one mouse.

H– K Acquired resistance in ALL-50.
Complementary data to experiment depicted in Figure **1J**.

H Quantification of imaging signals complementing Figure **1J. I**ndividual mice of single treatment groups, untreated mice and all remaining mice not included in Figure 1C are shown.

I Proportion of PDX ALL-50 cells in murine BM was determined and is depicted as boxplot with median, 25th and 75th percentile and min/max indicated by whiskers; each dot represents one mouse; colors as defined in Figure **1A**. ***: $p < 0.001$ by one-way ANOVA followed by Tukey's multiple comparisons test.

J Correlation of imaging signals (Figure **1J** and **S1F**) and PDX proportion in murine BM as determined by flow cytometry (Figure S**1G**); each dot represents one mouse. Correlation curve and R^2 were calculated using non-linear regression.

K **Acquired resistance in ALL-265.** Experiment was performed as described in Figure **1A,C,** except that ALL-265 was used and mice were treated with 0.3 mg/kg VCR and 70 mg/kg Cyclo. Quantification of bioluminescence imaging (BLI) signals is shown. Each line represents one mouse for VCR (n=2), Cyclo (n=2), and combination treatment (n=19). Dashed line indicates treatment period.

Figure S2

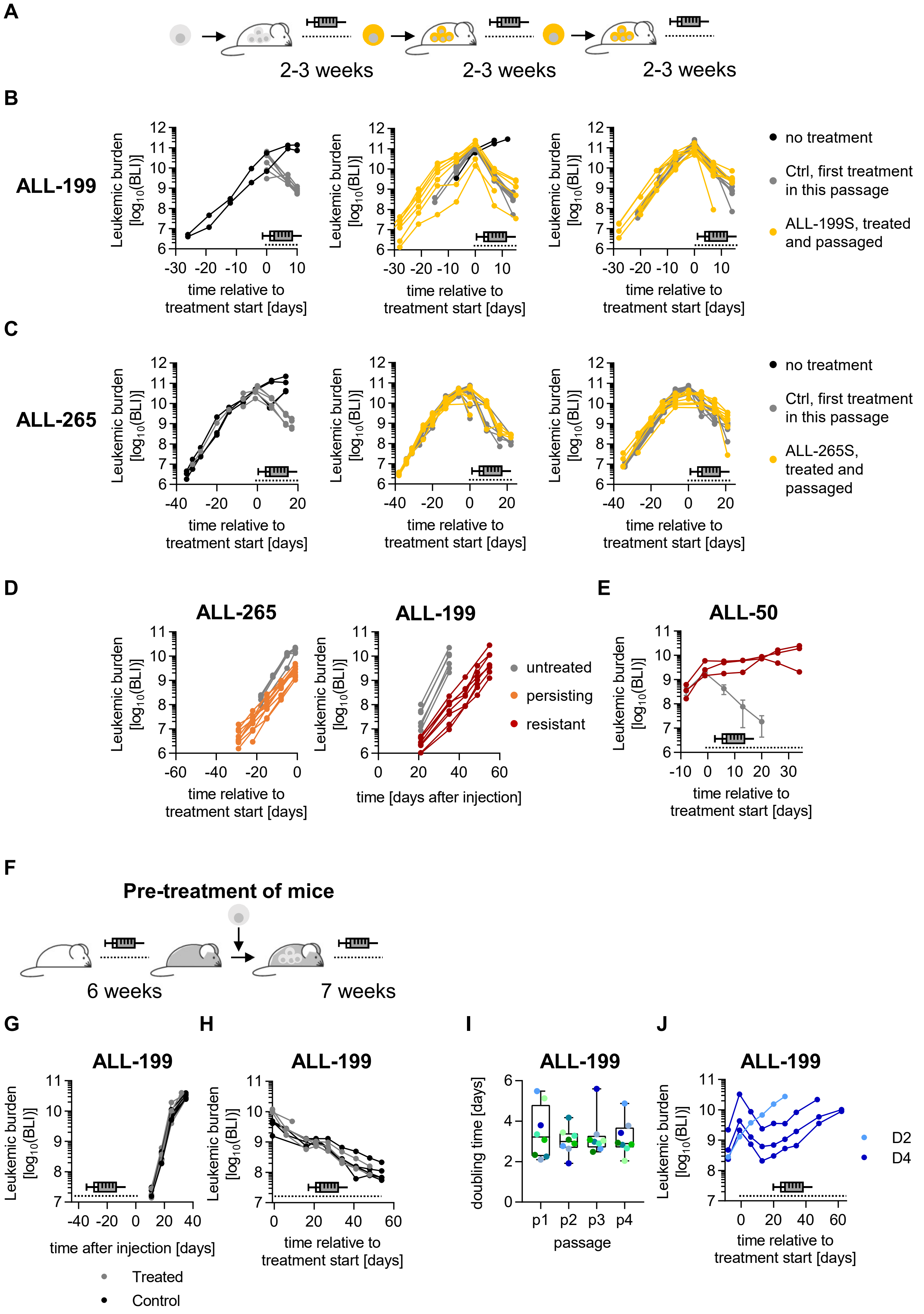
A
2-3 weeks
2-3 weeks
2-3 weeks
B
ALL-199
Leukemic burden [log10(BLI)]
time relative to treatment start [days]
no treatment
Ctrl, first treatment in this passage
ALL-199S, treated and passaged
C
ALL-265
Leukemic burden [log10(BLI)]
time relative to treatment start [days]
no treatment
Ctrl, first treatment in this passage
ALL-265S, treated and passaged
D
ALL-265
ALL-199
Leukemic burden [log10(BLI)]
time relative to treatment start [days]
time [days after injection]
untreated
persisting
resistant
E
ALL-50
Leukemic burden [log10(BLI)]
time relative to treatment start [days]
F
Pre-treatment of mice
6 weeks
7 weeks
G
ALL-199
Leukemic burden [log10(BLI)]
time after injection [days]
Treated
Control
H
ALL-199
Leukemic burden [log10(BLI)]
time relative to treatment start [days]
I
ALL-199
doubling time [days]
p1
p2
p3
p4
passage
J
ALL-199
Leukemic burden [log10(BLI)]
time relative to treatment start [days]
D2
D4

Supplementary Figure S2. Functional characterization of PDX ALL at distinct stages following treatment start. Complementary data to Figure 2

A-C Re-transplantation preserves sensitive phenotype.

Experimental procedure (**A**): In a first passage (left panel) ALL-199 (**B**) or ALL-265 (**C**) cells were engrafted into NSG mice and either treated (grey; n=8 ALL-199, n=6 ALL-265)) for 2 or 3 weeks with combination chemotherapy (treatment was performed as described in Fig. 1 except that 0.5 mg/kg VCR and 100 mg/kg Cyclo were used) or were left untreated (black, n=2 ALL-199, n=6 ALL-265). Cells were isolated and re-transplanted into secondary recipient mice and treated again with the identical treatment regimen (middle panels, yellow, n=8 for ALL-199, n=9 for ALL-265,) or left untreated (black, n=2 for ALL-199). The procedure was repeated for a third passage (right panels, yellow, n=8). In parallel, previously untreated cells of ALL-199 (**B**) or ALL-265 (**C**) were engrafted and treated with the same treatment regimen, serving as controls (middle and right panels, grey, n=8 for ALL-199, n=9 for middle panel of ALL-265, n=8 for right panel of ALL-265). Growth was monitored by repetitive imaging and imaging signals were quantified and are plotted for each passage. Data of middle panel of ALL-199 is also shown in Fig. 2B; each line represents one mouse. Dashed line and the syringe symbol indicate treatment period.

D Analysis of growth in secondary recipient mice. Quantification of imaging signals used to calculate doubling time of ALL-265 and ALL-199 in Figure **2C** and **2D** is shown.

E Analysis of treatment response of ALL-50R in secondary recipient mice. Experiment was set up exactly as described in Figure **2D** except that ALL-50R (red, n=3) and adjusted drug doses (0.25 mg/kg VCR and 70 mg/kg Cyclo) were used. Data from untreated ALL-50 cells (grey, mean +/- SD of n=20 mice) derived from experiment described in Figure **1J** as included for comparison. Dashed line indicates treatment period.

F- H Pre-treatment of mice does not influence cell growth or treatment response.

Experimental procedure (**F**): ALL-199 cells were injected into NSG mice which were either pre-treated with combination chemotherapy (n=10, treatment was performed as described in Fig. 1, VCR 0.15 mg/kg, Cyclo 70 mg/kg) for 6 weeks before cell injection or into control mice without pre-treatment (n=10). N=5 mice per group were injected with low cell numbers and engraftment and growth was monitored (**G**) by imaging. N=5 mice per group were injected with high cell numbers and at high leukemia burden, all mice were treated with combination chemotherapy (treatment was performed as described in Fig. 1, VCR 0.15 mg/kg, Cyclo 70 mg/kg) for 7 weeks. Engraftment and growth (**G**) and treatment response (**H**) was monitored by imaging. Each line represents one mouse.

I – J Resistance remains stable despite prolonged drug holiday. Complementary data to Figure 2E.

I Doubling times of resistant ALL-199 derivatives D1-D8 were calculated based on imaging during serial passaging as shown in Figure 2E. Each dot represents one mouse, each color indicates one derivative. One-way ANOVA followed by Tukey´s multiple comparisons test revealed no significant difference.

J Combination Chemotherapy of the 4th passage was repeated with additional mice harboring ALL-199 D4 (n=3) and D2 (n=1) cells. Each line represents one mouse, each color indicates one derivative. Dashed line indicates treatment period.

Figure S3

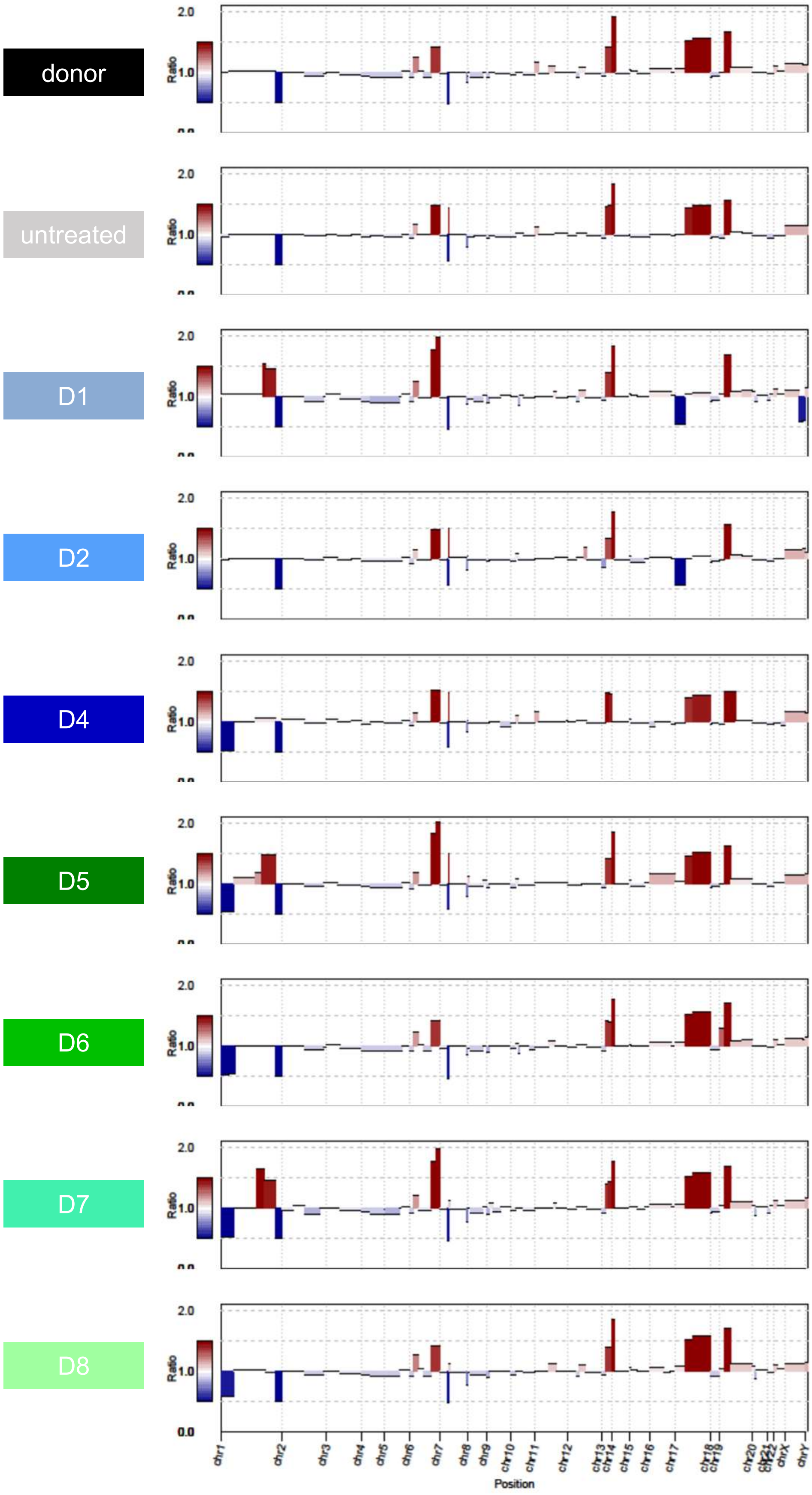

Supplementary Figure S3. CNAs of resistant derivatives of ALL-199. Complementary data to Figure 3A-E.

CNA of donor ALL-199 PDX cells, untreated ALL-199U and resistant ALL-199R derivatives as determined by whole exome sequencing. Losses compared to germ line control are depicted in blue and gains in red. Cut-offs for a copy number segment: min. size 5Mb, max. five segments per Chromosome, cut-off for CNA call <0.8 or >1.2, respectively.

Figure S4

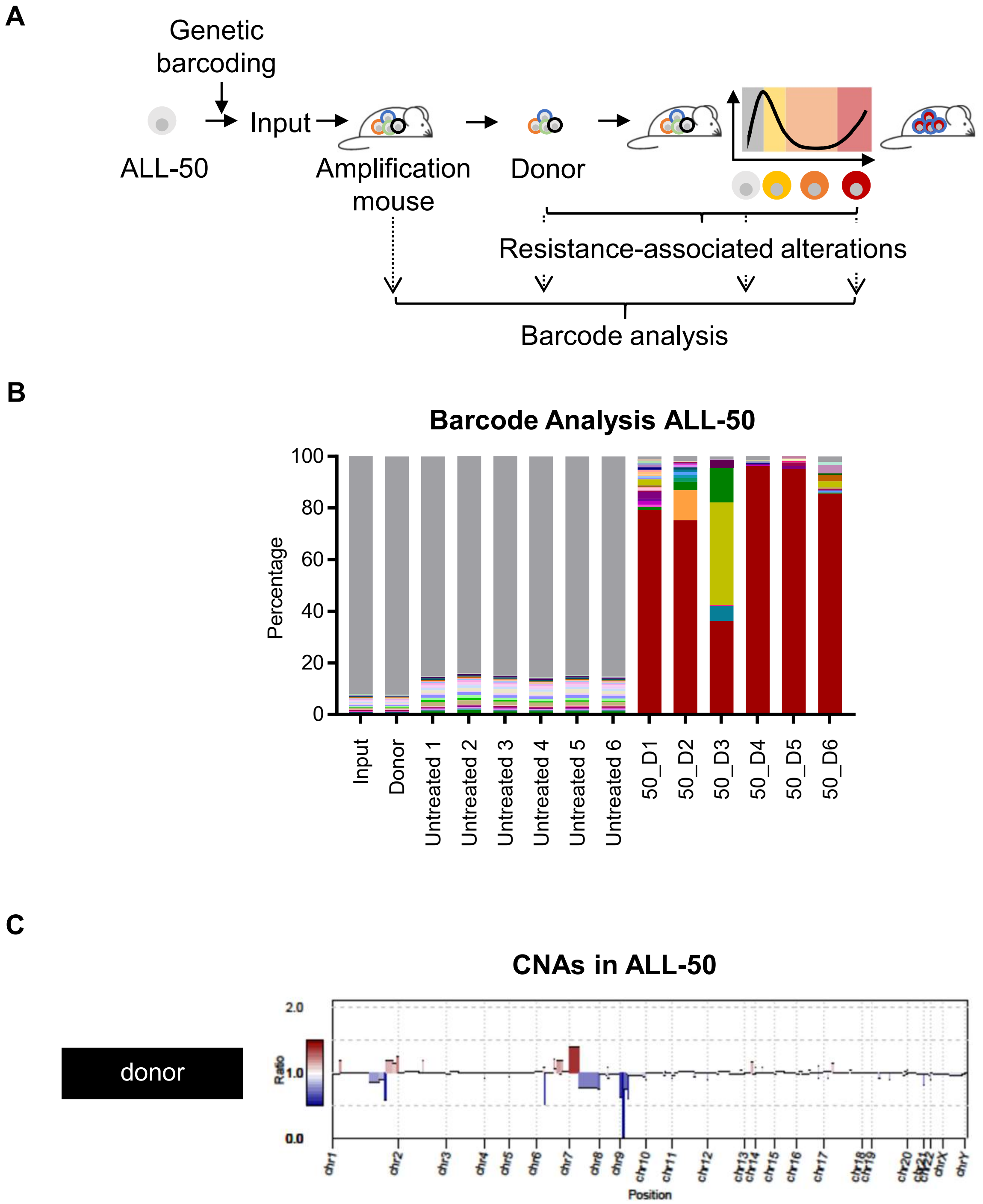
A
Genetic barcoding
ALL-50
Input
Amplification mouse
Donor
Resistance-associated alterations
Barcode analysis
B
Barcode Analysis ALL-50
Percentage
100
80
60
40
20
0
Input
Donor
Untreated 1
Untreated 2
Untreated 3
Untreated 4
Untreated 5
Untreated 6
50_D1
50_D2
50_D3
50_D4
50_D5
50_D6
C
CNAs in ALL-50
donor
2.0
1.0
0.0
Ratio
Position

Figure S4

D

Resistance-associated alterations of ALL-50

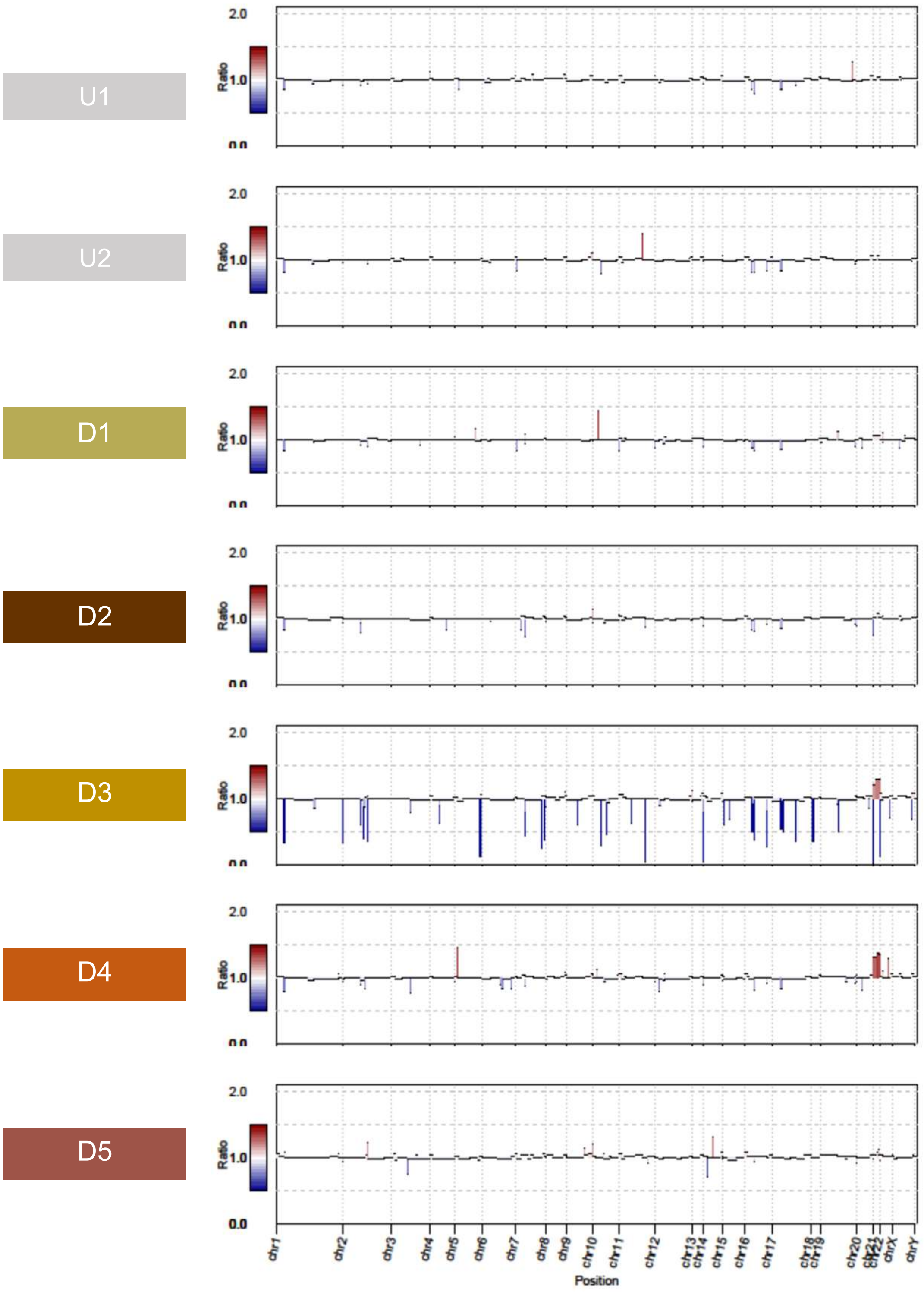

Supplementary Figure S4. Genomic characterization of resistant derivatives of ALL-50
Complementary data to Figure 3.

A Experimental procedure. ALL-50 PDX cells were marked so that each clone received an individual barcode. Cells were amplified in mice for 5 weeks; the resulting population of $5*10^6$ cells contained approximately 750 different barcodes with an average of 700 cells per barcode. Aliquots of identically barcoded cells were re-transplanted into mice and mice were subjected to long-term chemotherapy with results shown in Figure 1J. Mice were sacrificed at defined time points. Barcode and whole exome sequencing was performed.

B Barcode analysis. Transduced ALL-50 cells before injection into mice (Input); barcoded ALL-50 cells following amplification (Donor) and cells from six ALL-50U and six ALL-50R mice from experiments described in Figure 1J were analyzed for barcode distribution. Each color represents an individual genetic barcode corresponding to an individual leukemic cell marked before transplantation into the amplification mice, while grey indicates the sum of all barcodes/ clones present at a frequency lower than 0,5%.

C-D CNAs of untreated and resistant derivatives of ALL-50.

CNA of donor ALL-50 PDX cells compared to primary patient leukemia sample (C) and CNA of ALL-50 U1, U2 and ALL-50 D1-D6 cells compared to donor (D) as determined by whole exome sequencing. Losses compared to germ line control are depicted in blue and gains in red. Cut-offs for a copy number segment: min. size 100kb, max. five segments per Chromosome. Data of D6 had to be excluded due to poor sequencing quality.

Figure S5

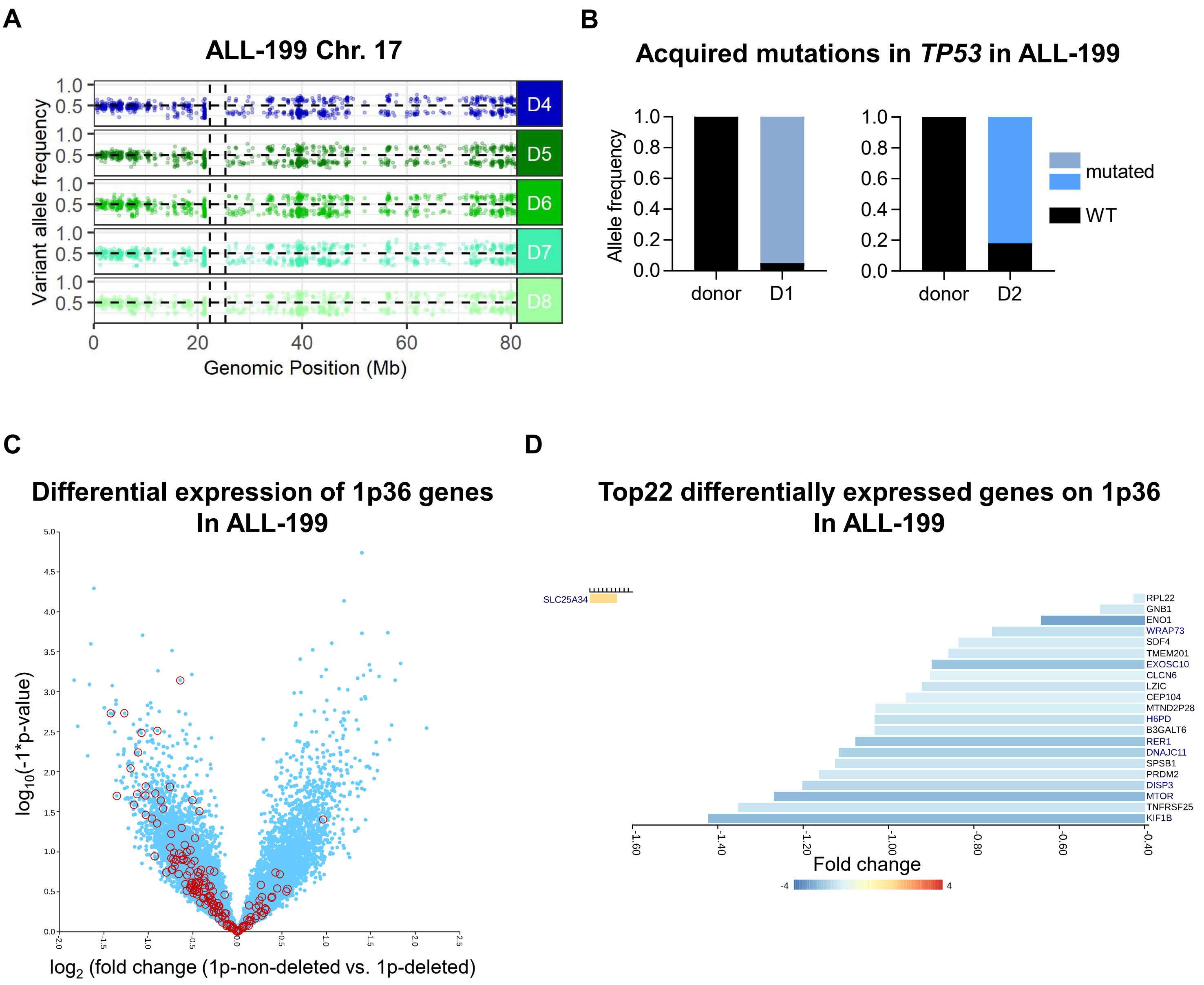

Supplementary Figure S5. SNV and transcriptome analysis of ALL-199.
Complementary data to Figure 3E-G.

A Remaining data of chr 17 SNV analysis of ALL-199 derivatives not included in Figure 3E are shown.

B VAF of the two different acquired SNV in TP53 in D1 and D2; see also Table S3.

C Volcano plot of genes differentially expressed in 1p-non-deleted (ALL-199 D1 and D2) vs 1p-deleted (ALL-199 D4-D8) derivatives. Genes mapping to the 1p36 smallest region of overlapping deletion (SRO) defined in derivatives D4-D8 are marked in red.

D Fold bar plot representing the top 22 most differentially expressed 1p36 SRO genes in ALL-199 1p-non-deleted vs 1p deleted derivatives.

Figure S6

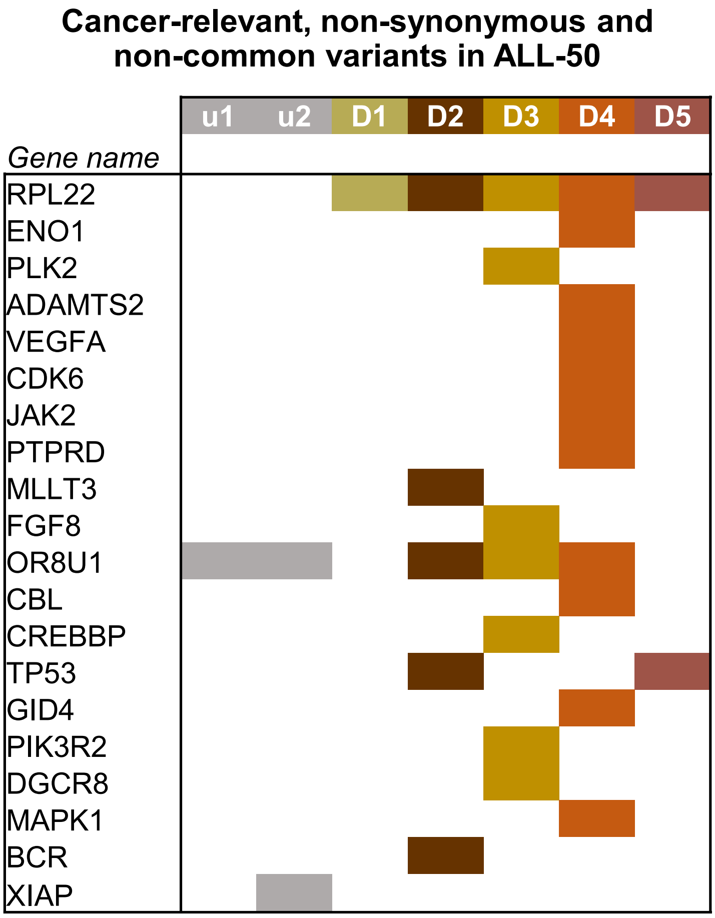

Supplementary Figure S6. Genomic characterization of resistant derivatives of ALL-50

Complementary data to Figure 3.

Cancer-relevant, non-synonymous and non-common variants of untreated and resistant ALL-50 PDX cells compared to donor ALL-50 PDX cells as determined by whole exome sequencing. Color indicates mutation in the respective gene in the respective sample. Mutations were sorted by genomic position. Details on mutations are provided in supplementary Table S5.

Figure S7

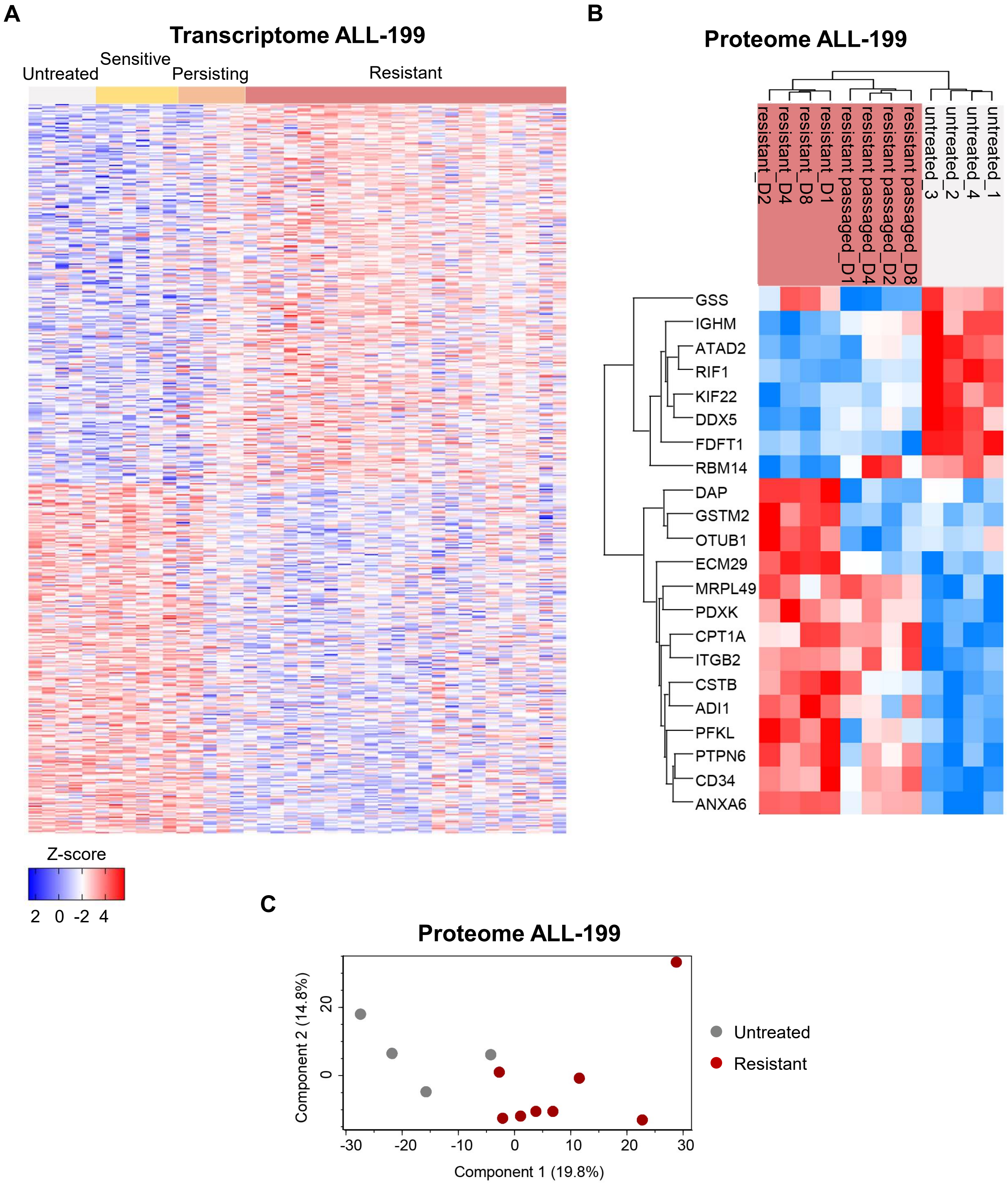

Supplementary Figure S7. Transcriptomic and proteomic profiling of PDX ALL-199 cells at distinct stages of in vivo treatment. Complementary data to Figure 3G-I

- **A** Experiment described in Figure 3H. Heatmap of 525 differentially expressed genes with $p < 0.01$ between ALL-199U and ALL-199R is shown. Gene list is provided in Table S4.
- **B** Experiment described in Figure 3I. Heatmap of top 22 differentially expressed proteins between ALL-199U and ALL-199R is shown. Protein list is provided in Table S5
- **C** Principal component analysis of proteome data. Each dot represents one mouse.

Figure S8

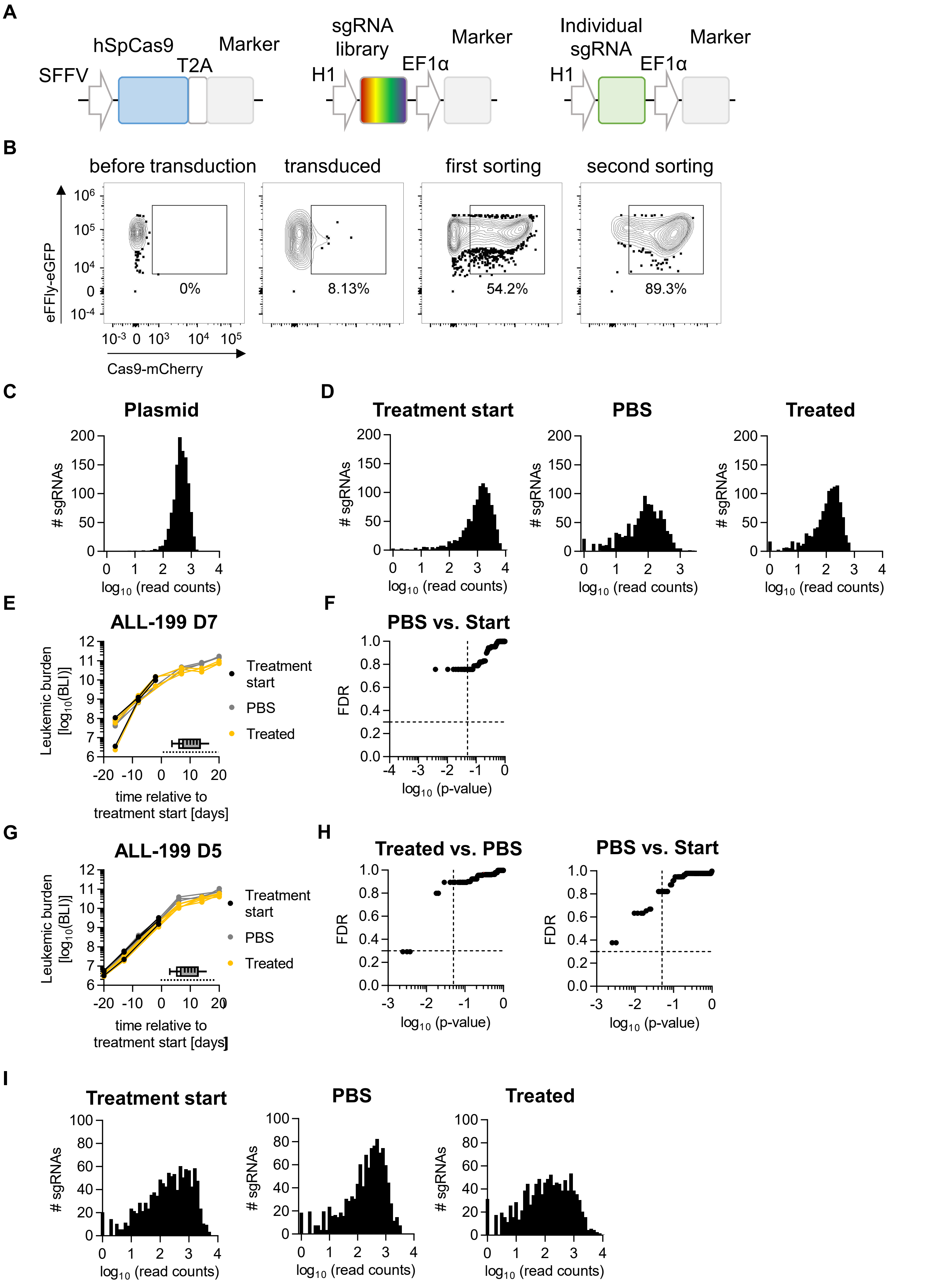
A
SFFV
hSpCas9
T2A
Marker
H1
sgRNA library
EF1α
Marker
H1
Individual sgRNA
EF1α
Marker
B
before transduction
transduced
first sorting
second sorting
eFFly-eGFP
Cas9-mCherry
0%
8.13%
54.2%
89.3%
C
Plasmid
sgRNAs
log10 (read counts)
D
Treatment start
PBS
Treated
E
ALL-199 D7
Leukemic burden [log10(BLI)]
time relative to treatment start [days]
Treatment start
PBS
Treated
F
PBS vs. Start
FDR
log10 (p-value)
G
ALL-199 D5
H
Treated vs. PBS
PBS vs. Start
I
Treatment start
PBS
Treated

Supplementary Figure S8: CRISPR/Cas9 screen of BCL2 in resistant ALL-199 derivatives. Complementary data to Figure 4

A Lentiviral constructs. Left: Expression of humanized codon-optimized S. pyogenes Cas9 (hSpCas9), linked to a marker (mCherry) via a 2A peptide and under control of the spleen focus-forming virus (SFFV) promoter. Middle and right: Expression of the sgRNA library (middle) or of an individual sgRNA (right) under control of the H1 promoter, linked to a marker (H2Kk-mtagBFP) under control of the EF1α promoter.

B Transduction and enrichment of ALL-199 D7 cells transgenic for the Cas9 construct. Flow cytometric analysis of Cas9 expression in resistant ALL-199 D7 before transduction, on day 7 after transduction and after two enrichment steps by flow cytometry.

C Read count distribution of sgRNA library plasmid after cloning as determined by NGS and counting of reads per individual sgRNA (log10 read counts per sgRNA).

D Read count distribution of one representative sample from experiment described in Figure 4A per indicated group and time point. sgRNA locus was amplified from purified PDX gDNA and analyzed by NGS.

E Quantification of imaging signals during in vivo engraftment and treatment from experiment described in Figure 4A. One line represents one mouse. Dashed line indicates treatment period.

F MAGeCK comparison of PBS vs. start from experiment described in Figure 4A. Plotted is p-value vs. false discovery rate (FDR) for each individual comparison. One dot represents one gene (pooled analysis of 5 sgRNAs per gene). Dashed lines indicate cut-off of $p = 0.05$ and FDR = 0.3.

G Experiment described in Figure 4A was repeated except that ALL-199 D5 cells were used. Quantification of imaging signals during in vivo engraftment and treatment. One line represents one mouse (n=3 treatment start, n=3 PBS, n=5 treated). Dashed line indicates treatment period.

H MAGeCK comparison of treated vs. PBS (upper panel) and PBS vs. start (lower panel) from experiment described in Figure S5G. Plotted is p-value vs. FDR for each individual comparison. One dot represents one gene (pooled analysis of 5 sgRNAs per gene). Dashed lines indicate cut-off of $p = 0.05$ and FDR = 0.3. See Table S7 for detailed gene list.

I Read count distribution of one representative sample per indicated group from experiment described in Figure S5G. sgRNA locus was amplified from purified PDX gDNA and analyzed by NGS.

Figure S9

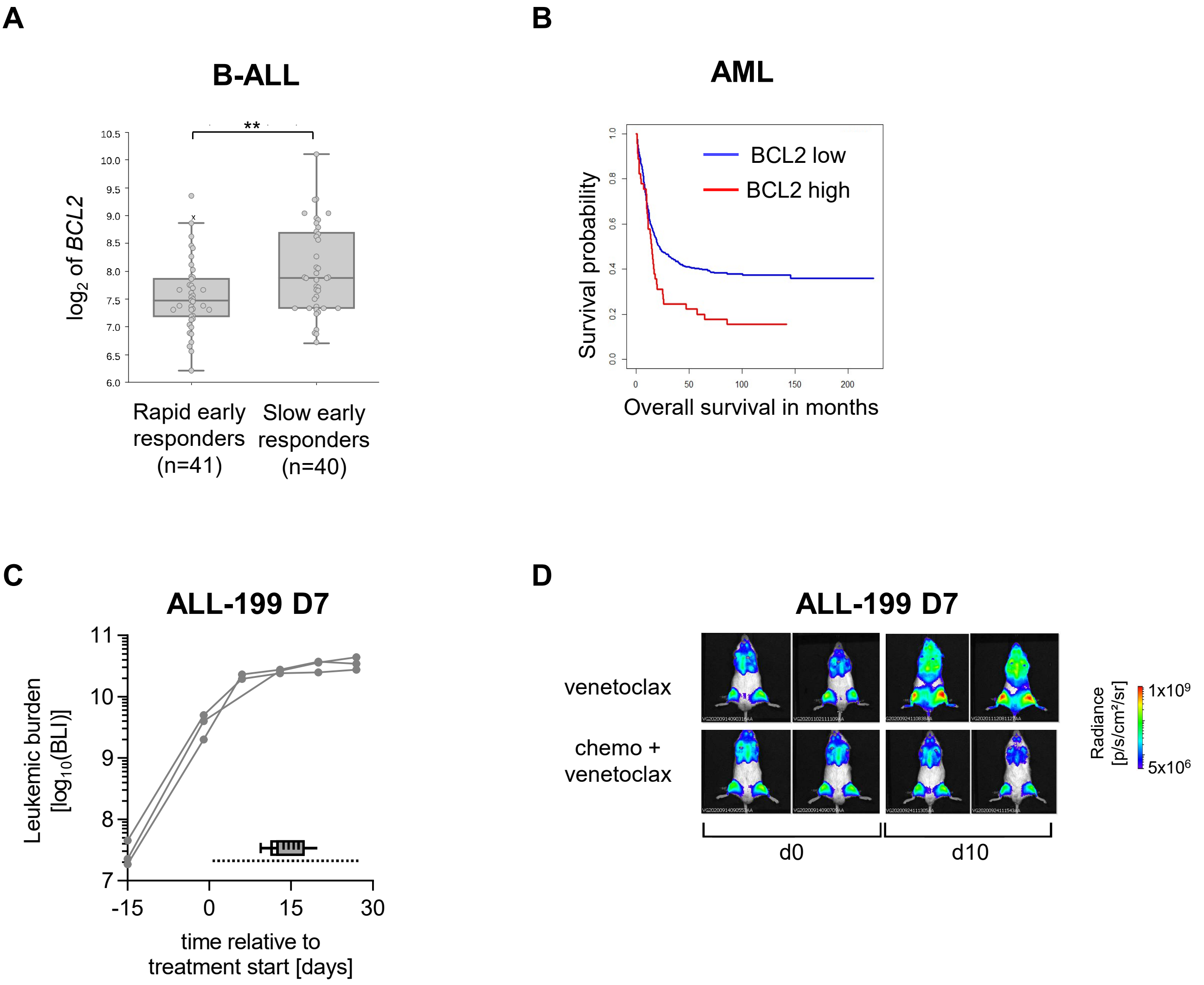

Supplementary Figure S9: BCL2 inhibition sensitizes resistant derivatives to chemotherapy. Complementary data to Figure 4

A Higher BCL2 mRNA expression correlated with slow early response. BCL2 mRNA expression in diagnostic samples of children with high-risk B-precursor ALL, comparing patients with rapid (M1 marrow, <5% blasts, n=41) and slow (M3 marrow, >25% blasts, n=40) early response at day 7 towards a standard four-drug induction treatment ($p<0.01$, t-test, data were re-analyzed from the publicly available dataset GSE7440).

B High BCL2 mRNA expression is prognostically unfavorable in AML. Reanalysis of a publicly available microarray dataset consisting of 449 AML patients treated in different trials of the Haemato-Oncology Foundation for Adults in the Netherlands (HOVON). The data were part of a publicly available cohort analyzed by Affymetrix (GSE14468). The association between gene expression and overall survival was analyzed with the Cox proportional hazards model. The expression of the BCL2 gene was significantly associated with poor overall survival (HR = 1.71, p = 0.0055). Cut-off for high BCL2 expression was defined by the top 10% of BCL2 expression, all remaining samples were defined as BCL2 low.

C Individual mice of control group shown in Figure 4E. One line represents one mouse harbouring ALL-199 D7 cells with non-targeting control sgRNA. Dashed line indicates treatment period.

D Imaging picture of two more representative mice engrafted with ALL-199 D7 cells from experiment described in Figure 4GH per group at d0 and d10 of treatment.
